# Cascaded-mode interferometers: Spectral shape and linewidth engineering

**DOI:** 10.1126/sciadv.adt4154

**Published:** 2025-03-19

**Authors:** Jinsheng Lu, Ileana-Cristina Benea-Chelmus, Vincent Ginis, Marcus Ossiander, Federico Capasso

**Affiliations:** ^1^ Harvard John A. Paulson School of Engineering and Applied Sciences, 9 Oxford Street, Cambridge, MA 02138, USA.; ^2^ Hybrid Photonics Laboratory, École Polytechnique Fédérale de Lausanne (EPFL), CH-1015 Lausanne, Switzerland.; ^3^ Data Lab/Applied Physics, Vrije Universiteit Brussel, 1050 Brussels, Belgium.; ^4^ Institute of Experimental Physics, Graz University of Technology, 8010 Graz, Austria.

## Abstract

Interferometers are essential tools for measuring and shaping optical fields, widely used in optical metrology, sensing, laser physics, and quantum mechanics. They superimpose waves with a mutual phase delay, modifying light intensity. A frequency-dependent phase delay enables spectral shaping for filtering, routing, wave shaping, or multiplexing. Conventional Mach-Zehnder interferometers generate sinusoidal output intensities, limiting spectral engineering capabilities. Here, we propose a framework that uses interference of multiple transverse modes within a single multimode waveguide to achieve arbitrary spectral shapes in a compact geometry. Designed corrugated gratings couple these modes, enabling energy exchange akin to a beam splitter for easy multimode handling. We theoretically and experimentally demonstrate spectra with independently tunable linewidth and free spectral range, along with distinct spectral shapes for various transverse modes. Our method applies to orthogonal modes of different orders, polarization, and angular momentum, offering potential for sensing, calibration, metrology, and computing.

## Teaser

A framework using interference of multiple transverse modes in a single waveguide compactly achieves arbitrary spectral shapes.

## INTRODUCTION

The manipulation and control of the amplitude and phase of broadband light at each wavelength, known as optical spectral shaping, is fundamental for applications such as pulse shaping (*1*–*7*), microwave waveform generation through wavelength-to-time mapping of optical signals (*8*–*16*), and sensing in biochemistry, medicine, and physics (*17*–*23*). The first attempt to manipulate the optical spectrum dates back to Newton’s prism experiments (*24*), where white light was decomposed into its constituent colors. Building on Newton’s work, researchers have then implemented a spatial mask or spatial light modulator to control the amplitude (and possibly the phase) of each of these colors (*1*–*5*). This parallel manipulation offers spectral control with a frequency resolution limited by the pixel size of the mask and the beam diameter at the mask and requires large components and space, making miniaturization challenging.

**Fig. 1.**
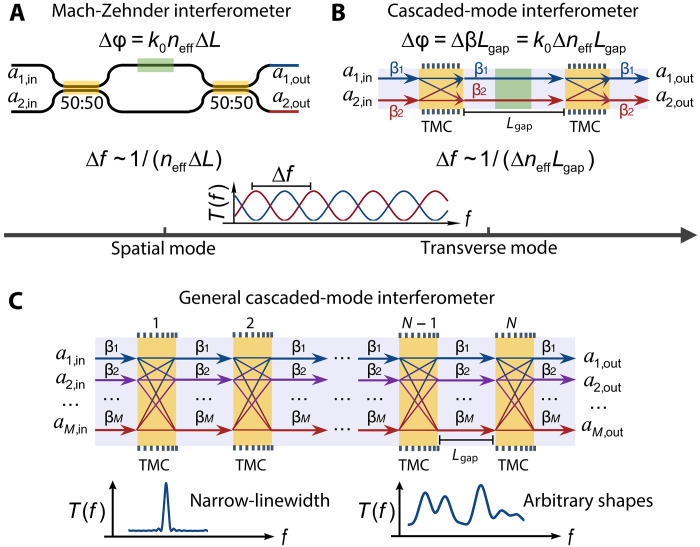
Concept of cascaded-mode interferometers. (**A**) A typical Mach-Zehnder interferometer (MZI) with two inputs and two outputs. The relative phase shift between the two arms is $\Delta \varphi =k_{0}n_{\text{eff}}\Delta L$, where $k_{0}$, $n_{\text{eff}}$, and ΔL are the wave number, the effective index of the spatial mode, and the length difference of the two arms, respectively. The output interference spectrum is shown below and its free spectral range (Δf) is proportional to $1/\left(n_{\text{eff}}\Delta L\right)$. Note that the group index $n_{g}$ should be used here if dispersion is considered. (**B**) A cascaded-mode interferometer, as a counterpart to the MZI. It consists of two orthogonal modes (propagation constants $\beta_{1}$ and $\beta_{2}$) in a multimode waveguide and two TMCs separated by a distance $L_{\text{gap}}$. The relative phase shift between the two modes is $\Delta \varphi =k_{0}\Delta n_{\text{eff}}L_{\text{gap}}$, where $\Delta n_{\text{eff}}$ is the effective index difference of the modes. Its free spectral range of the output spectrum is proportional to $1/\left(\Delta n_{\text{eff}}L_{\text{gap}}\right)$. Orthogonal modes can include examples such as the linearly polarized (LP) modes in multimode optical fibers or the quasi-transverse electric modes in photonic integrated circuits. (**C**) A general cascaded-mode interferometer, where multiple orthogonal modes with propagation constants $\beta_{j}$ are converted and mixed by multiple TMCs separated by $L_{\text{gap}}$. $a_{j,\text{in}}$ and $a_{j,\text{out}}$ (j=1,2,…,M) represent the amplitudes of input and output mode j, respectively. A narrow linewidth spectrum and a spectrum with arbitrary shapes generated by suitably designed cascaded-mode interferometers are shown below.

Simple filtering functions can be implemented via a simple Mach-Zehnder interferometer (Fig. 1A) (*25*) that splits and recombines two beams (denoted as $a_{1,\text{in}}$ and $a_{2,\text{in}}$) after sending them along two paths that differ by a length ΔL. Such interferometers are routinely implemented in integrated photonics, using on the platform’s ability to realize compact splitters and waveguides of arbitrary length. However, the spectral response depends sinusoidally on frequency. Consequently, the output power spectrum of the two output beams (denoted as $a_{1,\text{out}}$ and $a_{2,\text{out}}$) oscillates with a periodicity $\Delta f\propto 1/\left(n_{\text{eff}}\Delta L\right)$ that depends on the effective index $n_{\text{eff}}$ of the waveguide mode and the path length difference ΔL (we do not consider dispersion here for simplicity). Bragg gratings provide finer control that achieves wavelength-specific and bandwidth-controlled reflection or filtering through interference of an infinity of waves (*26*, *27*). Such narrow-linewidth response relies strictly on invoking more than two waves and can also be achieved using multilayer thin films (*28*–*30*), Fabry-Perot interferometers (*25*), arrayed waveguide gratings (*31*), and fiber interferometers (*32*–*35*). More complex spectral manipulation can be achieved using on-chip spectral shapers, typically consisting of multiple resonators, which offer high spectral resolution and programmability (*8*–*11*, *36*, *37*).

The working principle of most optical devices mentioned above relies on the interference of beams that are reflected multiple times. However, their amplitudes are constrained by the reflection or transmission coefficients of the mirrors or interfaces, and the phases are limited by the propagation lengths and propagation constants, which are integer multiples of the cavity length or thin-film thickness. This typically results in the amplitudes being dependent on each other, leaving the requirement for independent control unaddressed. Furthermore, the propagation constants of these beams are typically the same.

In this work, we propose to shape the spectra of light by using an alternative to conventional Mach-Zehnder interferometers: We exploit multiple transverse modes of a multimode waveguide on silicon-on-insulator (SOI) platform, instead of the spatial modes of two individual waveguides (Fig. 1A). To couple these modes, we use transmissive mode converters (TMCs) that transfer energy from one mode to another, depending on the so-called splitting ratio (SR), similar to a beam splitter. The working principle of the TMC is based on long-period gratings that satisfy the Bragg condition for coupling between different modes. This approach enables a similar spectral shaping when using two TMCs in a geometry as shown in Fig. 1B, albeit with a periodicity that is not determined by a path imbalance but instead by an imbalance in the propagation constants ($\beta_{i}=\frac{2\pi }{\lambda }n_{\text{eff},i}$, i=1,2) of the two modes $\Delta f\propto 1/\left(\Delta n_{\text{eff}}L_{\text{gap}}\right)$, with $\Delta n_{\text{eff}}$ being the difference in the effective refractive index of the used transverse modes and $L_{\text{gap}}$ being the length of the multimode waveguide between the mode converters. We show that this compact implementation provides a straightforward extension to cascading more mode converters (N) and a higher number of modes (M) with propagation constants $\beta_{1},\beta_{2},\ldots ,\beta_{M}$ (Fig. 1C). Building upon this concept, we develop and present a generalized framework that computes the exact spectra of multiple interfering transverse modes through transfer matrix formalism and their dependency on the SR of the mode converters. We demonstrate narrow-linewidth (i.e., high finesse) and arbitrary spectra using our cascaded-mode interferometers. One of the promising aspects of our device is that, unlike traditional technologies such as Fabry-Perot interferometers, its finesse remains unaffected by losses, enabling the integration of switchable or active materials without compromising the device’s spectral performance.

## RESULTS

### Spectral engineering using cascaded-mode interferometers

In the case of N TMCs spaced by the same distance $L_{\text{gap}}$ (Fig. 1C), the amplitudes of the modes at the output of the interferometer depend on the input modes and the transfer function of the various components as

$$a_{\text{out}}\left(\lambda \right)=T_{c}{\left(T_{\text{wg}}T_{c}\right)}^{N-1}a_{\text{in}}$$ (1)

where $a_{\text{in}}={\left(a_{1,\text{in}},a_{2,\text{in}},\ldots ,a_{M,\text{in}}\right)}^{T}$ and $a_{\text{out}}={\left(a_{1,\text{out}},a_{2,\text{out}},\ldots ,a_{M,\text{in}}\right)}^{T}$ are the amplitude arrays of the input and output modes of the interferometer. This is only true for pure forward-scattering converters. $T_{c}$ and $T_{\text{wg}}$ are the transmittance matrix of the mode converter and the multimode waveguide between the mode converters, respectively. The mode j accumulates a phase term $H_{j}\left(\lambda \right)=e^{-i\beta_{j}L_{\text{gap}}}=e^{-i\frac{2\pi }{\lambda }n_{\text{eff},j}L_{\text{gap}}}$ (j=1,2,…,M) during propagation in the multimode waveguide between the mode converters. Therefore, $T_{\text{wg}}$ represents a propagation phase matrix, which can be written as

$$T_{\text{wg}}=\left[\begin{array}{cccc} H_{1}\left(\lambda \right) & 0 & \ldots & 0\\ 0 & H_{2}\left(\lambda \right) & \ldots & 0\\ \ldots & \ldots & \ldots & \ldots \\ 0 & 0 & \ldots & H_{M}\left(\lambda \right) \end{array}\right]$$ (2)

The transmittance matrix $T_{c}$ representing an arbitrary mode conversion (or so-called beam splitting) function is given by

$$T_{c}=\left(\begin{array}{cccc} t_{11} & t_{12} & \ldots & t_{1M}\\ t_{21} & t_{22} & \ldots & t_{2M}\\ \ldots & \ldots & \ldots & \ldots \\ t_{M1} & t_{M2} & \ldots & t_{\mathrm{MM}} \end{array}\right)$$ (3)

where $t_{\mathrm{ij}}$ (i,j=1,2,…,M) is the transmittance coefficient indicating the mode conversion from mode j to mode i in a transmissive way. $T_{c}$ and $T_{\text{wg}}$ are unitary matrices if there is no loss. $T_{c}$ is a symmetric matrix if the mode conversion is reciprocal. The two matrices ($T_{c}$ and $T_{\text{wg}}$) applied sequentially in Eq. 1 resemble the evolution matrices of the adiabatic impulse model, which is used to describe qubit dynamics (*38*, *39*).

We consider a case that N=2 to show the ability of arbitrary spectral shaping of the cascaded-mode interferometer. Equation 1 can then be simplified to $a_{\text{out}}=T_{c}T_{\text{wg}}T_{c}a_{\text{in}}$. Combined with Eqs. 2 and 3, the amplitude spectrum of mode j in the output of the cascaded-mode interferometer can be calculated as

$$a_{j,\text{out}}\left(\lambda \right)=\sum_{m=1}^{M}\sum_{n=1}^{M}t_{\mathrm{jn}}e^{-i\frac{2\pi }{\lambda }n_{\text{eff},n}L_{\text{gap}}}t_{\mathrm{nm}}a_{m,\text{in}}$$ (4)

Note that the effective index $n_{\text{eff},n}$ in this series is not freely selectable but is instead restricted to specific values, typically nonequidistant, determined by the waveguide cross section. Despite these nonequidistant $n_{\text{eff},n}$ values, the series can still effectively approximate a wide range of predetermined functions, similar to a standard Fourier series (*40*). Therefore, by designing the transmittance coefficients $t_{\mathrm{ij}}$, we can achieve nearly arbitrary spectral shapes of the output modes.

### On-chip cascaded-mode interferometers

**Fig. 2.**
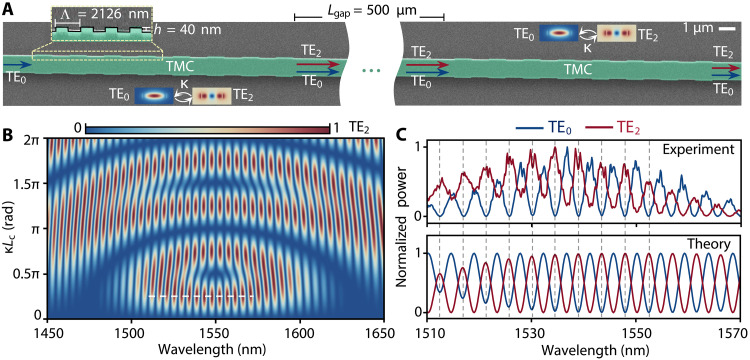
Interference spectra generated by a cascaded-mode interferometer. (**A**) Scanning electron microscope image of a fabricated cascaded-mode interferometer comprising two waveguide transverse modes (TE_0_ and TE_2_) and two TMCs separated by a distance $L_{\text{gap}}=500$ μm. The interferometer is fabricated from 220-nm–thick SOI material. The TMCs are made of gratings with period Λ=2126 nm, the length $L_{c}=8\Lambda =17$ μm, and the corrugated grating depth h=40 nm. The width of the multimode waveguide is 1100 nm. The aspect ratio of the zoomed-in image in (A) is set to 1:6 to visualize the gratings better. (**B**) Calculated output power spectra of the TE_2_ modes for varying coupling strength $\kappa L_{c}$ which is the product of the grating’s coupling coefficient (κ) and the grating length ($L_{c}$). The white dashed lines in (B) correspond to the red curve (theory) in (C) where $\kappa L_{c}=0.25\pi$, that is, a mode power SR of 50:50. (**C**) Measured and calculated output power spectra of the TE_0_ and TE_2_ modes when $L_{\text{gap}}=500$ μm and $\kappa L_{c}=0.25\pi$. The input mode is TE_0_ mode.

In the first experiment, we realize the most simple on-chip cascaded-mode interferometer, featuring two input modes and two output modes that we choose to be TE_0_ and TE_2_ as shown in Fig. 2A. The interferometer is fabricated from 220-nm–thick SOI material (see Materials and Methods for details). We corrugate the multimode waveguide with a periodicity of Λ and a depth of h, satisfying the Bragg condition: $\beta_{1}-\beta_{2}-m\frac{2\pi }{\Lambda }=0$. We use the first order m=1. This grating provides a momentum at the central wavelength $\lambda_{0}$ to satisfy the phase matching condition ($\Lambda =\lambda_{0}/\Delta n_{\text{eff}}$), enabling codirectional coupling between the TE_0_ and TE_2_ mode. It uses long periods. In contrast, distributed-feedback laser gratings, which use short periods, function as mirrors by reflecting waves backward through contradirectional coupling. The coupling coefficient in both codirectional and contradirectional coupling scenarios is influenced by similar factors, such as grating corrugation depth, waveguide geometry, and mode profile. For identical waveguide geometry and corrugation depth, the coupling coefficients will be comparable in both scenarios (*41*). The transmittance coefficients $t_{\mathrm{ij}}$ (i,j=1,2) in the transmittance matrix $T_{c}$ of this mode converter can be derived from coupled mode theory (*42*) (section S1) as $t_{11},t_{22}=\text{cos}\left({\mathrm{sL}}_{c}\right)\mp i\frac{\delta }{s}\text{sin}\left({\mathrm{sL}}_{c}\right)$ and $t_{12}=t_{21}=-i\frac{\kappa }{s}\text{sin}\left({\mathrm{sL}}_{c}\right)$, where $\delta =\frac{\pi \Delta n_{\text{eff}}}{\lambda }-\frac{\pi }{\Lambda }$ is the phase mismatch, κ is the coupling coefficient, $L_{c}$ is the grating length, and $s=\sqrt{\delta^{2}+\kappa^{2}}$. Phase matching is only achieved at the central wavelength, that is, $\delta \left(\lambda =\lambda_{0}\right)=0$, and the phase mismatch is proportional to the deviation of the wavelength from the central wavelength: $\delta \left(\lambda \right)\approx \frac{\pi \Delta n_{\text{eff}}\left(\lambda -\lambda_{0}\right)}{\lambda_{0}^{2}}$. The power conversion efficiency of the mode converter is $\eta =\frac{\kappa^{2}}{\kappa^{2}+\delta^{2}}{\text{sin}}^{2}\left({\mathrm{sL}}_{c}\right)$. The bandwidth of the mode converter ($\Delta \lambda_{\text{BW}}$), determined by this term $\frac{\kappa^{2}}{\kappa^{2}+\delta^{2}}$, can be derived as $\Delta \lambda_{\text{BW}}=\frac{2{\kappa \lambda }_{0}^{2}}{\pi \Delta n_{\text{eff}}}$, which increases with κ (section S4 and fig. S24). Suppose we input TE_0_ mode into this cascaded-mode interferometer, that is, $a_{\text{in}}={\left[1,0\right]}^{T}$, the amplitude of the TE_0_ mode at the output can be calculated using Eq. 4 as

$$\begin{array}{c} a_{1,\text{out}}={\left[\text{cos}\left({\mathrm{sL}}_{c}\right)-i\frac{\delta }{s}\text{sin}\left({\mathrm{sL}}_{c}\right)\right]}^{2}\\ e^{-i\frac{2\pi }{\lambda }n_{\text{eff},1}L_{\text{gap}}}-\frac{\kappa^{2}}{s^{2}}{\text{sin}}^{2}\left({\mathrm{sL}}_{c}\right)e^{-i\frac{2\pi }{\lambda }n_{\text{eff},2}L_{\text{gap}}} \end{array}$$ (5)

When the phase mismatch is small compared to the coupling coefficient, δ≪κ, Eq. 5 simplifies to $a_{1,\text{out}}={\text{cos}}^{2}\left(\kappa L_{c}\right)e^{-i\frac{2\pi }{\lambda }n_{\text{eff},1}L_{\text{gap}}}-$ ${\text{sin}}^{2}\left(\kappa L_{c}\right)e^{-i\frac{2\pi }{\lambda }n_{\text{eff},2}L_{\text{gap}}}$. The power of the TE_2_ mode can be calculated as ${|a_{2,\text{out}}|}^{2}=1-{|a_{1,\text{out}}|}^{2}$ according to energy conservation; the power of the TE_0_ mode, ${|a_{1,\text{out}}|}^{2}$, can be determined from Eq. 5. Alternatively, as derived in detail in section S4, ${|a_{2,\text{out}}|}^{2}$ can be expressed as

$${|a_{2,\text{out}}|}^{2}=2\eta \left(1-\eta \right)\left(1+\text{cos}\Delta \phi \right)$$ (6)

where the phase difference Δϕ is given by $\Delta \phi =\frac{2\pi \left(n_{\text{eff},1}-n_{\text{eff},2}\right)L_{\text{gap}}}{\lambda }+$$2\text{arctan}\text{[}\frac{\delta }{s}\text{tan}\left({\mathrm{sL}}_{c}\right)\text{]}$ and η is the power conversion efficiency (see above). The left term, 2η(1−η), is an amplitude modulator, which generates envelope patterns, such as the doughnut-shaped structures observed in the power spectra (see Fig. 2B and fig. S24D). The second term, 1+cosΔϕ, introduces fine structures, including spectral interference patterns and fork-shaped features (see Fig. 2B and fig. S24E). Together, the equation captures the interplay of coupling, detuning, and phase matching, which govern the power distribution between the coupled modes in a cascaded-mode interferometer (see also section S4).

To achieve a sinusoidal modulation of the power transmitted through the interferometer as a function of frequency with maximal visibility (for normalized power, this means the power modulation spans the full range from 0 to 1), the two TMCs need to split the power equally into TE_0_ and TE_2_, in analogy to 50:50 beam splitters used in conventional Mach-Zehnder modulators (fig. S7). Therefore, the coupling strength $\kappa L_{c}$ should be equal to $\frac{\pi }{4}$, leading to $a_{1,\text{out}}=\frac{1}{2}\left(e^{-i\frac{2\pi }{\lambda }n_{\text{eff},1}L_{\text{gap}}}-e^{-i\frac{2\pi }{\lambda }n_{\text{eff},2}L_{\text{gap}}}\right)$ (when δ≪κ) and a maximal visibility of the interference spectrum.

**Fig. 3.**
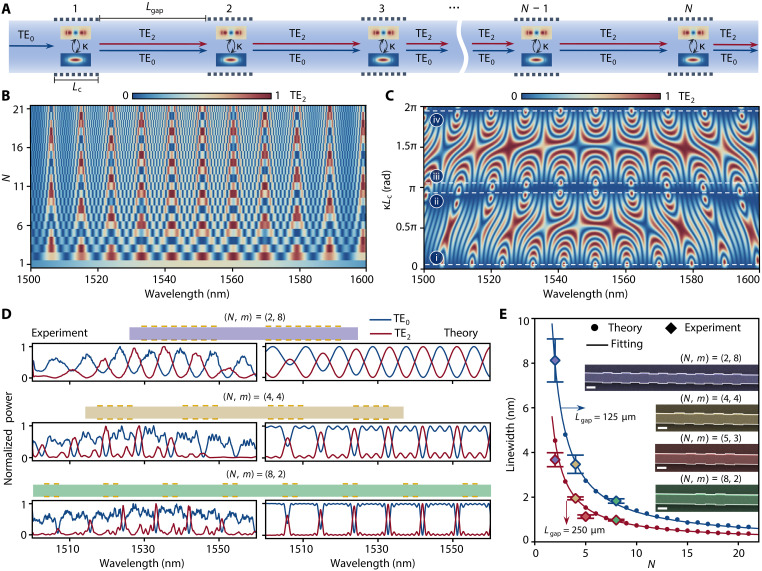
Cascaded-mode interferometer with multiple TMCs for precise tuning of FSR and linewidth. (**A**) Schematic of a cascaded-mode interferometer with multiple TMCs and two modes (TE_0_ and TE_2_). (**B**) Calculated output power spectra of the TE_2_ mode with varying the number (N) of TMCs at $\kappa L_{c}=0.25\pi$. Parameters used in the numerical calculations: $L_{\text{gap}}=250$μm, $a_{1,\text{in}}=1$, $a_{2,\text{in}}=0$, $n_{1,\text{eff}}=2.74$, and $n_{2,\text{eff}}=1.98$. (**C**) Calculated output power spectra of the TE_2_ mode for varying coupling strength $\kappa L_{c}$ when N=8. The four dashed lines in (C) represent the positions where (i) $\kappa L_{c}=0.5\pi /N$, (ii) $\kappa L_{c}=\left(N-0.5\right)\pi /N$, (iii) $\kappa L_{c}=\left(N+0.5\right)\pi /N$, and (iv) $\kappa L_{c}=\left(2N-0.5\right)\pi /N$ with N=8. (**D**) Measured (left) and calculated (right) output power spectra of the two modes for different N with coupling strengths $\kappa L_{c}=0.5\pi /N$ (N=2,4,8). The multiplication of the number of the mode converters N and the number of the grating period of a single mode converter m equals 16. (**E**) Measured and calculated linewidth variation with N. Fitting equations: 12.88/(N−0.38) and 7.02/(N−0.44) for $L_{\text{gap}}=125$ μm and $L_{\text{gap}}=250$ μm, respectively. The input mode is TE_0_ mode. The inserts are the scanning electron microscope images of the mode converters of the fabricated cascaded-mode interferometer device at different N. The scale bars are 1 μm.

**Fig. 4.**
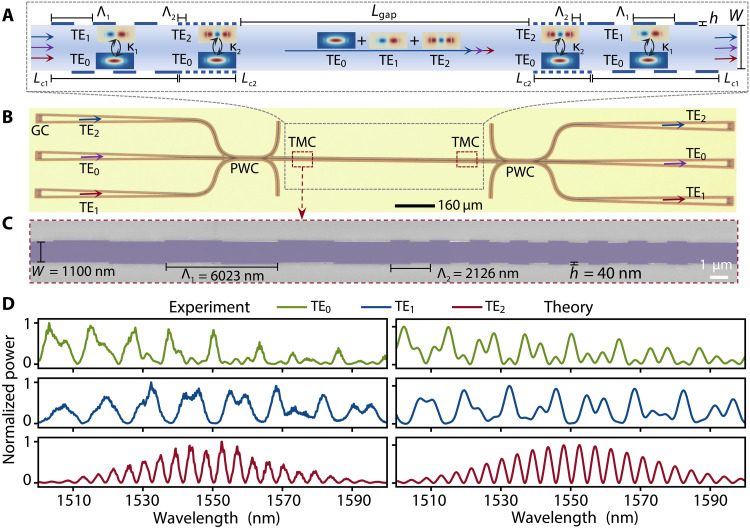
Cascaded-mode interferometer with multiple waveguide modes for parallel spectra engineering. (**A**) Schematic of a cascaded-mode interferometer with three waveguide modes (TE_0_, TE_1_, and TE_2_) and two TMCs separated by a distance of $L_{\text{gap}}$. The TMCs consist of two sets of gratings. One set of the grating is asymmetric and with the period $\Lambda_{1}=6023$ nm, the length $L_{c1}=3\Lambda =18$ μm, and the coupling strength $\kappa_{1}L_{c1}=0.19\pi$, used for mode conversion between TE_0_ and TE_1_. The other set of grating is symmetric and with the period $\Lambda_{2}=2126$ nm, the length $L_{c2}=8\Lambda =17$ μm, and the coupling strength $\kappa_{2}L_{c2}=0.25\pi$, used for mode conversion between TE_0_ and TE_2_. The multimode waveguide width is W=1100 nm. $L_{\text{gap}}=500$ μm. The grating depth is 40 nm. (**B**) Optical image of the fabricated device. GC, grating coupler; PWC, parallel waveguide coupler; TMC, transmissive mode converter. (**C**) Scanning electron microscope image of the left TMC in (B). (**D**) Measured and calculated output power spectra of TE_0_, TE_1_, and TE_2_ modes when the input mode is TE_0_ mode.

Figure 2B depicts the calculated wavelength-dependent transmitted power contained in mode TE_2_ after the interferometer upon sending TE_0_ into the interferometer, as a function of coupling strength $\kappa L_{c}$ for a gap length $L_{\text{gap}}=500$ μm. We notice that the lowest coupling strength for maximal visibility is when $\kappa L_{c}=\frac{\pi }{4}$ (white dashed cut line). Operating the cascaded-mode interferometer at this point allows to use the shortest length for the mode converter, which is beneficial for the footprint of the device or, alternatively, the smallest corrugation. The bandwidth of the mode converter is limited by phase mismatch, which becomes detrimental as soon as δ becomes a substantial portion of s. As follows from Eq. 5, the bandwidth can be increased by increasing the coupling strength to, for example, $\kappa L_{c}=\frac{3\pi }{4}$ or $\kappa L_{c}=\frac{5\pi }{4}$. In these cases, the larger coupling strength compensates for the phase mismatch, although at the expense of longer gratings or larger corrugations. To validate our concept, we experimentally report the wavelength-dependent power of the output modes TE_0_ and TE_2_ after the interferometer, under an input TE_0_ mode (upper graph of Fig. 2C). We use a grating period Λ=2126 nm. The coupling coefficient of the mode conversion between the TE_0_ and TE_2_ modes is $\kappa =\frac{\pi }{32\Lambda }=0.046$ μm^−1^. We observe alternating powers that match well with our analytical model (lower graph of Fig. 2C and fig. S7) as well as simulation results (fig. S8), in line with expectations from a Mach-Zehnder interferometer. Note that the envelope of the measured spectra in Fig. 2C (also in Figs. 3D and 4D) results from the parallel waveguide coupler (section S2 and fig. S5), which is used to load and unload the modes from the multimode waveguide. The free spectral range of the interference spectra is measured as 19.4, 8.5, and 4.4 nm when $L_{\text{gap}}=100$, 250, and 500 μm, respectively, which are in good agreement with the calculations: 21.5, 8.6, and 4.3 nm using the formula $\Delta \lambda_{\text{FSR}}=\frac{\lambda_{0}^{2}}{\left(n_{g,1}-n_{g,2}\right)L_{\text{gap}}}$, where $n_{g,1}=4.85$ and $n_{g,2}=3.75$ are the group indexes of the TE_0_ and TE_2_ modes at the central wavelength $\lambda_{0}=1538$ nm (fig. S9). The group indexes were obtained from numerical simulations and verified in a previous work (*43*).

In many optical applications, controlling light’s spectrum to achieve a narrower transmission linewidth is desirable, for example, in filtering or routing. In the following, we will show that cascading several mode converters provide a useful knob to achieve this, as visualized in Fig. 3A. A tempting approach could be to simply cascade N 50:50 mode converters along a single multimode waveguide. However, we find from computing the total transfer function of the system using Eq. 1 that concatenating N 50:50 mode converters leads to a narrowing of the transmission spectra, at the expense of multiple undesired sidebands (Fig. 3B and fig. S10). This is typically not desired in filtering and routing applications that rely on achieving a vanishingly small insertion loss only at one desired wavelength and close to zero transmission elsewhere. Guided by our mathematical derivation (section S3), we find that, instead, the optimal coupling strength of the grating needs to generally satisfy the condition $\kappa L_{c}=\frac{\pi }{2N}$, with N being the total number of mode converters. In this case, the corresponding SR of the mode converter at the central wavelength is $\text{SR}=\frac{\eta }{1-\eta }$, where $\eta ={\text{sin}}^{2}\left(\frac{\pi }{2N}\right)$. For N=8, the mode converter has an SR of around 0.04, which means that when 1 mW TE_0_ mode is input into the mode converter, it gets 0.038 mW TE_2_ mode and 0.962 mW TE_0_ mode. We exemplify this by reporting in Fig. 3C the total transmitted power for TE_2_ and N=8 for various coupling strengths $\kappa L_{c}$. As expected, at $\kappa L_{c}=\frac{\pi }{2\times 8}$, the transmitted power features narrow-linewidth transmission [dashed line (i)]. Other coupling strengths where this is satisfied are $\kappa L_{c}=\pi \pm \frac{\pi }{2N}$, $\kappa L_{c}=2\pi \pm \frac{\pi }{2N}$, and so on (N=8).

We fabricated a series of cascaded-mode interferometers with N=2,4,and8 while adjusting the number of periods (*m*) to satisfy $\kappa L_{c}=\frac{\pi }{2N}$ for each one of them. We can easily get $m\times N=\frac{\pi }{2\kappa \Lambda }=16$ according to $L_{c}=m\Lambda$ and $\kappa =\frac{\pi }{32\Lambda }$. Consequently, the number of the grating periods of the mode converter is m=8,4,and2, respectively. The maximum achievable N is 8 in this case (because m×N=16 and m≥2). However, by using a multimode waveguide with a larger width or a mode conversion grating with weaker corrugation, we can reduce the coupling coefficient (κ), thereby enabling a substantially larger N. For example, the maximum achievable N becomes 34 when the width of the multimode waveguide and the corrugation depth of the grating are 2500 and 20 nm, respectively (fig. S23). Note that in the condition of $\kappa L_{c}<\frac{\pi }{2N}$, we can still obtain narrow linewidth spectra but with a smaller amplitude at the power spectrum peaks, which is determined by ${|a_{\text{peak}}|}^{2}={\text{sin}}^{2}\left(\kappa L_{c}N\right)$ (fig. S23).

In line with our modeling, we experimentally find that an increased number of converters markedly reduce the linewidth of the transmission spectra while efficiently suppressing its off-resonance transmission, as shown in Fig. 3D. We find the experimental linewidth to match well with theory, which is corroborated by two sets of devices with different gap lengths $L_{\text{gap}}=125$ μm (fig. S15) and $L_{\text{gap}}=250$ μm (Fig. 3D). The free spectral range of the narrow linewidth spectra generated by this cascaded-mode interferometer, in this case, can be decreased by increasing the gap between TMCs $\left(\Delta \lambda_{\text{FSR}}\propto \frac{1}{L_{\text{gap}}}\right)$ (fig. S14), and the linewidth (full width at half maximum) $\Delta \lambda_{\text{FWHM}}$ decreases when increasing the number N of TMCs (Fig. 3 and figs. S11 to S13), which can be approximated by $\frac{\Delta \lambda_{\text{FSR}}}{N}$. A more accurate expression would be $\Delta \lambda_{\text{FWHM}}=\frac{\Delta \lambda_{\text{FSR}}+c_{1}}{N+c_{2}}$, where $c_{1}$ and $c_{2}$ are fitting coefficients (Fig. 3E). The finesse of this cascaded-mode interferometer, defined as the ratio of the free spectral range and the linewidth, therefore, is approximately equal to the number of the mode converters: $F=\frac{\Delta \lambda_{\text{FSR}}}{\Delta \lambda_{\text{FWHM}}}\approx N$. We note here, however, that, unlike in the case of resonators, the finesse is not related to a field enhancement, but rather to the contrast of the transmission spectrum in a given band. In contrast to the finesse of a traditional Fabry-Perot interferometer that is sensitive to loss, the finesse of our cascaded-mode interferometer is loss independent, whereas the total transmitted power is loss dependent (fig. S22).

### Multidimensional on-chip cascaded-mode interferometers

Shaping the spectrum of a light source simultaneously in multiple ways is a requirement in many applications. For example, having the ability to flatten part of the spectrum can be beneficial for spectroscopy over a broad bandwidth, and simultaneously modulating another part of it in intensity can be important for achieving more complex time-domain profiles. A large $\frac{\Delta \lambda_{\text{FSR}}}{\Delta \lambda_{\text{FWHM}}}$ is also often needed to cut fundamental radiation in spectroscopy or on-chip generated frequency combs.

We show in the following that such manipulation of light’s spectrum can be accomplished by extending the cascaded-mode interferometer of Fig. 2 to provide efficient conversion between not only two but more distinct transverse modes. In this case, the spectral profile of two orthogonal modes can be arbitrarily shaped by dumping the remaining energy into the third mode (figs. S16 to S21). To exemplify this concept, we resort to mode converters that feature a pair of periodicities, as to convert TE_0_ into TE_1_ with a coupling strength $\kappa_{1}$ and TE_0_ into TE_2_ with a coupling strength $\kappa_{2}$, as seen in Fig. 4A. Figure 4B depicts an optical microscope image of the fabricated device, showcasing straightforward multiplexing and demultiplexing of the various transverse modes using mode-selective parallel waveguide couplers (section S2 and figs. S5 and S6). Coupling TE_0_ with TE_1_ requires a grating that is asymmetrically displaced on the two sides of the waveguide due to the different symmetry of the two modes, whereas coupling TE_0_ with TE_2_ requires a symmetric one, as visible from the scanning electron microscope image in Fig. 4C. By choosing $\kappa_{1}L_{c1}=0.19\pi$ and $\kappa_{1}L_{c1}=0.25\pi$, we experimentally confirm in Fig. 4D (left) (also in fig. S18) that substantially different spectral shapes can be achieved for the three transverse modes, in line with our analytical description (Fig. 4D, right).

## DISCUSSION

In summary, we demonstrate how the interference of multiple transverse modes in a single interferometer can be used to control the spectral response of light using TMCs. Our approach leverages the capabilities of photonics to engineer the effective index and propagation constant through waveguide design, and the conversion bandwidth and efficiency through corrugation. By propagating multiple modes with different propagation constants within a single compact multimode waveguide, we circumvent the need for multiple waveguides that are otherwise used in multiarmed interferometers. By designing index-matched parallel waveguide couplers, we multiplex and demultiplex these transverse modes. Altogether, this enables a smaller footprint and greater design flexibility than the traditional Mach-Zehnder interferometer.

Potential applications span a wide range from versatile tools for exploring complex physical phenomena to innovative nanophotonic devices for communication and sensing. Examples of applications are expected to emerge across three key aspects.

The cascaded-mode interferometer developed in this work provides an adaptable platform for nanophotonic sensing applications. By enabling narrow-linewidth transmission peaks and valleys tunable via mode converters, the system supports the detection of environmental changes such as temperature, strain, and chemical composition with advanced sensitivity compared with previous interferometric fiber optic sensors (*44*). Our cascaded-mode interferometer works in a transmissive way without reflections. Therefore, the light energy is distributed in the entire device, which differs from the Fabry-Perot resonators where energy is built up in the cavity. This approach offers robustness to optical loss and improved reliability for distributed optical sensing over large-scale systems (*45*). These attributes provide enhanced flexibility and reliability compared to localized sensing technologies, supporting compact and integrated designs for sensing applications (*46*).

This approach also facilitates fundamental physical studies of phenomena associated with the interference effects between multiple transverse modes, polarization states (*47*), and angular momentum modes in integrated circuits or optical fibers (*48*). The ability to integrate nonlinear optical effects, such as second-harmonic generation and supercontinuum generation, with mode converter integrated photonics, provides opportunities to investigate wave interactions and mode coupling dynamics in nonlinear optical systems.

In addition, the cascaded-mode interferometer offers potential applications in quantum interference studies and integrated photonic communications. Our approach is more flexible in spectral shaping compared to alternative solutions proposed using transmissive Bragg gratings on the silicon nitride platform (*49*), or multiarmed interferometers in fibers (*50*). As such, it can become a tool for scalable on-chip quantum interference between many modes (*51*, *52*), contributing to the development of quantum computing and communication technologies. The ability to achieve precise spectral shaping, including customizable free spectral ranges and tunable linewidths, addresses challenges in wavelength-division multiplexing, spectral filtering, and waveform engineering for optical communications. Adding programmability to the device via thermo- or electro-optical tunable mode converters supports reconfigurable photonic circuits and space-efficient integrated optical computing applications (*53*–*56*).

Our studies offer a generalized theory framework for spectral shaping, opening up exciting directions for advanced sensing, wavelength-isolation filtering, waveform shaping, and narrow-linewidth light amplifying. The flexibility and compact design of the system expand the potential for interference-based optical engineering and inspire further developments in photonics, quantum technologies, and nonlinear optics.

## MATERIALS AND METHODS

### Sample fabrications

We use SOI material (thickness of the silicon device layer: 220 nm) to fabricate the cascaded-mode interferometers. The fabrication processes are as follows: First, the ZEP520A e-beam resist with a thickness of 450 nm is spin coated on the SOI substrate. Second, we use electron-beam lithography to write the designed structures and immerse after-exposure samples into O-xylene to develop the e-beam resist. Third, reactive-ion etching is used to etch the silicon device layer with a full etching depth of 220 nm, and Remover PG is used to remove all remaining resists. After that, a silicon oxide layer with 700 nm thickness is deposited on top of the devices as a protection layer using chemical vapor deposition.

### Numerical simulations

We use the finite-difference time-domain (FDTD) and the variational FDTD method (Ansys/Lumerical) to simulate and design our devices, including the grating couplers, parallel waveguide couplers, TMC gratings, and cascaded-mode interferometers. In simulations, the size parameters of the structures are used the same as in experiments. The refractive index of silicon and silicon oxide is 3.46 and 1.46, respectively.

### Optical measurements

The experiment measurement setup is shown in fig. S1. The laser source is a tunable Santec TSL-550 laser (tunable range: 1500 to 1630 nm, linewidth: 200 kHz, wavelength accuracy: ±3 pm). A fiber polarization controller is used to adjust the polarization of the light to reach the maximum coupling efficiency of the fiber-grating coupler, which is designed for transverse electric polarization. The output power is measured with an optical power meter Santec MPM-212 (power range: −80 to 10 dBm, wavelength range: 1250 to 1650 nm).

## Supplementary Materials

### This PDF file includes:

Supplementary Text Table S1 Figs. S1 to S24 References
