# Supplementary material for: Cascaded-mode interferometers: Spectral shape and linewidth engineering
Source: Sci Adv. 2025 Mar 19;11(12):eadt4154. doi: 10.1126/sciadv.adt4154 (PMC11922044; doi:10.1126/sciadv.adt4154)
Supplement: Supplementary file 1 — Supplementary Text Table S1 Figs. S1 to S24 References [file sciadv.adt4154_sm.pdf]

Science Advances
AAAS

Supplementary Materials for

Cascaded-mode interferometers: Spectral shape and linewidth engineering

Jinsheng Lu *et al.*

Corresponding author: Jinsheng Lu, jlu@seas.harvard.edu; Federico Capasso, capasso@seas.harvard.edu

Sci. Adv. **11**, eadt4154 (2025)
DOI: 10.1126/sciadv.adt4154

This PDF file includes:

Supplementary Text
Table S1
Figs. S1 to S24
References

1 Grating-assisted transmissive mode converter (TMC)

Coupled mode analysis

Here, we refer to the derivation of the coupled-mode theory from Huang (*42*). Consider two eigenmodes $\mathbf{E}_1 e^{i(\omega t-\beta_1 x)}$ and $\mathbf{E}_2 e^{i(\omega t-\beta_2 x)}$ with the propagation constants of β_1 and β_2 and the amplitudes of a_1 and a_2 inside a multimode waveguide.

These modes coexist and propagate independently inside the multimode waveguide since these eigenmodes are orthogonal. When the multimode waveguide is modified with periodic perturbation, that is, the grating (with the period Λ), coupling between these two modes happens, with a coupling coefficient κ positively correlates with the grating depth h (or the perturbation depth). The amplitudes of output modes and the input modes can be related to each other with the transfer matrix as

$$\mathbf{a}_{\text{out}} = \begin{pmatrix} a_1(L_c) \\ a_2(L_c) \end{pmatrix} = \mathbf{T}_c(L_c) \begin{pmatrix} a_1(0) \\ a_2(0) \end{pmatrix} = \mathbf{T}_c(L_c)\,\mathbf{a}_{\text{in}} \tag{S1a}$$

$$\mathbf{T}_c(L_c) = e^{-i\frac{\beta_1+\beta_2}{2}L_c} \begin{pmatrix} \cos(sL_c) - i\frac{\delta}{s}\sin(sL_c) & -i\frac{\kappa}{s}\sin(sL_c) \\ -i\frac{\kappa}{s}\sin(sL_c) & \cos(sL_c) + i\frac{\delta}{s}\sin(sL_c) \end{pmatrix} \tag{S1b}$$

$$\mathbf{T}_c(L_c) = e^{-i\frac{\beta_1+\beta_2}{2}L_c} \begin{pmatrix} \cos(\kappa L_c) & -i\sin(\kappa L_c) \\ -i\sin(\kappa L_c) & \cos(\kappa L_c) \end{pmatrix} \tag{S1c}$$

where L_c is the grating length. $\delta = \frac{1}{2}\left(\beta_1 - \beta_2 - \frac{2\pi}{\Lambda}\right)$ is the phase mismatch. Λ is the grating period. $s = \sqrt{\delta^2 + \kappa^2}$. $\mathbf{T}_c$ is the transmittance matrix of the grating-assisted mode converter. It is easy to prove that $\mathbf{T}_c(L_{c1} + L_{c2}) = \mathbf{T}_c(L_{c1})\,\mathbf{T}_c(L_{c2})$ with Eq. S1b. The transmittance matrix $\mathbf{T}_c$ can be further simplified, as shown in Eq. S1c when the phase matching condition is satisfied ($\delta = 0$). In this case, the mode conversion is mainly related to the coupling strength κL_c (multiplication of the coupling coefficient κ and the grating length L_c).

Theoretical calculations of the TMCs

Using the derivated transmittance matrix of the grating-assisted mode converter $\mathbf{T}_c$, we can theoretically calculate the amplitudes of the output modes for any given input. In the following, we consider two cases: (I) the transmissive mode converter consists of one set of gratings used for

two modes, and (II) the transmissive mode converter consists of two sets of gratings used for three modes.

(I) Single set of gratings for two modes. We use one set of gratings with the period of Λ_{12} to convert mode 1 to mode 2 (and vice versa), as shown in Fig. S2A. We use a silicon multimode waveguide with a width of 1100 nm and a thickness of 220 nm and consider two transverse electric modes, TE_0 and TE_2, in the multimode waveguide. The required grating period should be $\Lambda_{12} = \lambda_c/(\, n_{\text{eff},1} - n_{\text{eff},2})$ according to the momentum conservation principle during the mode conversion, where λ_c is the working center wavelength. When the input mode is the TE_0 mode ($a_{1,\text{in}} = 1$, and $a_{2,\text{in}} = 0$), according to Eq. S1, the power conversion efficiency from TE_0 to TE_2 should be

$$\eta = \left|a_{2,\text{out}}\right|^2 = \frac{1}{1 + (\delta/\kappa)^2} \sin^2\left(\kappa L_c \sqrt{1 + (\delta/\kappa)^2}\right) \tag{S2}$$

The output power ($|a_{\text{out}}|^2$) of the modes TE_0 and TE_2 when sweeping the coupling strength κ_c can be calculated using Eq. S2, as shown in Fig. S3. We can sweep the coupling strength through two ways: (I) vary L_c and keep κ constant (Fig. S3A, B); (II) vary κ and keep L_c constant (Fig. S3C, D). Note that the grating length L_c is related to the number of grating periods m by $L_c = \text{m}\Lambda_{12}$. Therefore, the grating length can only be varied discontinuously, while the coupling coefficient κ can be adjusted continuously by changing the grating depth h. However, a large grating depth can cause losses and significantly alter the original multimode waveguide, affecting the properties of its supported modes, such as their effective indices. In our experiments, we use a small grating depth (h = 40 nm) and achieve the desired coupling strength by adjusting the grating length L_c (or the number of grating periods m).

(II) Multiple sets of gratings for more than two modes. As shown in Fig. S2B, we use two sets of gratings (placed one after one) with the periods of Λ_{12} and Λ_{13}, the lengths of L_{c1} and L_{c2}, and the coupling coefficients of κ_1 and κ_2 to achieve mode conversion between the mode 1 and mode 2 and between the mode 1 and mode 3, respectively. For a silicon multimode waveguide with a width of 1100 nm and a height of 220 nm, it supports three TE modes (TE_0, TE_1, and TE_2), and the effective indexes of them at wavelength 1550 nm are $n_{\text{eff},1} = 2.74$, $n_{\text{eff},2} = 2.47$, and $n_{\text{eff},3} = 1.98$, respectively. The amplitude of the output modes $\mathbf{a}_{\text{out}}$ can be calculated using the transfer matrix formula as

$$\mathbf{a}_{\text{out}} = \mathbf{T}_{c2}\left(L_{c2}\right)\mathbf{T}_{c1}\left(L_{c1}\right)\mathbf{a}_{\text{in}} = \mathbf{T}_c\mathbf{a}_{\text{in}} \tag{S3}$$

The total transmittance matrix $\mathbf{T}_c$ for the three-mode TMC is calculated as:

$$\mathbf{T}_c = \mathbf{T}_{c2}\left(L_{c2}\right)\mathbf{T}_{c1}\left(L_{c1}\right) \tag{S4-a}$$

$$\mathbf{T}_{c1}\left(L_{c1}\right) = \begin{pmatrix} t_{11}^{c1} & t_{12}^{c1} & 0 \\ t_{21}^{c1} & t_{22}^{c1} & 0 \\ 0 & 0 & t_{33}^{c1} \end{pmatrix} \tag{S4-b}$$

$$\mathbf{T}_{c2}\left(L_{c2}\right) = \begin{pmatrix} t_{11}^{c2} & 0 & t_{13}^{c2} \\ 0 & t_{22}^{c2} & 0 \\ t_{31}^{c2} & 0 & t_{33}^{c2} \end{pmatrix} \tag{S4-c}$$

$$t_{11}^{c1} = \left(\cos\left(s_1 L_{c1}\right) - i\frac{\delta_1}{s_1}\sin\left(s_1 L_{c1}\right)\right) e^{-i\frac{\beta_1+\beta_2}{2}L_{c1}} \tag{S4-d}$$

$$t_{22}^{c1} = \left(\cos\left(s_1 L_{c1}\right) + i\frac{\delta_1}{s_1}\sin\left(s_1 L_{c1}\right)\right) e^{-i\frac{\beta_1+\beta_2}{2}L_{c1}} \tag{S4-e}$$

$$t_{12}^{c1} = t_{21}^{c1} = -i\frac{\kappa_1}{s_1}\sin\left(s_1 L_{c1}\right) e^{-i\frac{\beta_1+\beta_2}{2}L_{c1}} \tag{S4-f}$$

$$t_{33}^{c1} = e^{-i\beta_3 L_{c1}} \tag{S4-g}$$

$$t_{11}^{c2} = \left(\cos\left(s_2 L_{c2}\right) - i\frac{\delta_2}{s_2}\sin\left(s_2 L_{c2}\right)\right) e^{-i\frac{\beta_1+\beta_3}{2}L_{c2}} \tag{S4-h}$$

$$t_{33}^{c2} = \left(\cos\left(s_2 L_{c2}\right) + i\frac{\delta_2}{s_2}\sin\left(s_2 L_{c2}\right)\right) e^{-i\frac{\beta_1+\beta_3}{2}L_{c2}} \tag{S4-i}$$

$$t_{13}^{c2} = t_{31}^{c2} = -i\frac{\kappa_2}{s_2}\sin\left(s_2 L_{c2}\right) e^{-i\frac{\beta_1+\beta_3}{2}L_{c2}} \tag{S4-j}$$

$$t_{11}^{c1} = e^{-i\beta_1 L_{c2}} \tag{S4-k}$$

$$\delta_1 = \frac{\beta_1 - \beta_2}{2} - \frac{\pi}{\Lambda_1} \tag{S4-l}$$

$$\delta_2 = \frac{\beta_1 - \beta_3}{2} - \frac{\pi}{\Lambda_2} \tag{S4-m}$$

$$s_1 = \sqrt{\delta_1^2 + \kappa_1^2} \tag{S4-n}$$

$$s_2 = \sqrt{\delta_2^2 + \kappa_2^2} \tag{S4-o}$$

Simulations of the TMCs

To verify the theoretical results, we perform simulations for the TMC. In simulations, we use a multimode silicon waveguide with a width of 1100 nm and a thickness of 220 nm. The nano-gratings are corrugated on the sides of the multimode waveguide with a period of 2150 nm and a

corrugation depth of 40 nm. We input the TE_0 mode into this TMC. The simulated output power of the TE_0 and TE_2 modes varying with the grating length agrees with the theoretical results (Fig. S3 and S4).

2 Mode loading and unloading using parallel waveguide couplers

We use parallel waveguide couplers to load (Fig. S5A) or unload (Fig. S5B) modes in the multimode waveguide. The parallel waveguide couplers consist of three waveguides. The waveguide in the middle is a multimode waveguide (MWG) with a width of $W = 1100$ nm, supporting three transverse electric modes, TE_0, TE_1, and TE_2, whose mode profiles are shown in Fig. S6B. The waveguides on the sides are nanowaveguides used to load or unload the higher order modes (TE_1 and TE_2) to the multimode waveguide. To maximize the loading and unloading efficiency, the widths of the nanowaveguides on the sides need to be carefully designed to satisfy the phase matching condition. That is, the fundamental mode's effective index in the nanowaveguides must be equal to that of the higher order modes (TE_1 and TE_2) in the multimode waveguide. We simulate how the effective index of these modes (TE0, TE1, TE2, etc.) in a single waveguide change as the waveguide's width varies (Fig. S5C). We find that the width of the nanowaveguide 1 (NWG 1), which is used for loading and unloading the TE_2 mode, should be 534 nm. The width of the nanowaveguide 2 (NWG 2), which is used for loading and unloading the TE_1 mode, should be 336 nm (Fig. S5C).

To have a deeper understanding of the mode-coupling mechanism in the parallel waveguides, we solve the eigenmodes of a parallel waveguide (Fig. S6C) consisting of the multimode waveguide with a width of 1100 nm and the nanowaveguide with a width of 336 nm (Fig. S6D) or 534 nm (Fig. S6E). To distinguish the eigenmodes of this parallel waveguide from those of a single waveguide, we use TE'_i (where $i = 1, 2, 3$) to denote the transverse electric mode in this parallel waveguide structure. Interestingly, we find that there is a pair of symmetric and antisymmetric modes among the eigenmodes of the parallel waveguides (the TE'_2 and TE'_3 modes in Fig. S6D or the TE'_1 and TE'_2 modes in Fig. S6E). This pair of symmetric and antisymmetric modes in the parallel waveguides is approximately given by the sum and difference of the TE_0 mode in the nano waveguide and the high order mode in the multimode waveguide. Next, we examine how the effective indices of these modes in the parallel waveguide vary with the width of the nanowaveguide while keeping the width of the multimode waveguide fixed at 1100 nm (Fig. S6F and S6G). When the gap between the nanowaveguide and the multimode waveguide is relatively large (gap = 500 nm, as shown in Fig. S6G), we observe the effective index curve of the TE'_2 mode (red) almost intersects with that

of the TE_1' mode (black) and the TE_3' mode (blue) at a position where the nanowaveguide width is 336 nm and 534 nm, respectively. This observation aligns with the results in Fig. S5C, where we designed the nanowaveguide's width to match the effective index of the fundamental mode in the nanowaveguide with that of the higher-order modes in the multimode waveguide. When the gap between the nanowaveguide and the multimode waveguide is reduced to 200 nm (as shown in Fig. S6F), we observe anticrossing between these curves instead of an intersection. This anti-crossing phenomenon is caused by the stronger coupling between the nanowaveguide and the multimode waveguide when we reduce the gap between them, resulting in a larger difference in the effective indexes between the symmetric ($n_{\text{eff,symm}}$) and antisymmetric ($n_{\text{eff,anti}}$) modes at this position (where the nanowaveguide width is 336 nm or 534 nm).

Note that the light would be coupled back and forth between the multimode waveguide and the nano waveguide. We need to find the shortest coupling length (or called the critical coupling length L_{cc}) so that the energy of the TE_0 mode in the nano waveguide is completely coupled into the higher order mode (TE_1 or TE_2) in the multimode waveguide. The critical coupling length L_{cc} can be calculated using the effective indexes of the symmetric and antisymmetric mode ($n_{\text{eff,symm}}$ and $n_{\text{eff,anti}}$) as

$$L_{cc} = \frac{\lambda}{2\left(n_{\text{eff, symm}} - n_{\text{eff, anti}}\right)} \tag{S5}$$

As we mentioned above, a smaller gap between the nanowaveguide and the multimode waveguide results in a larger difference in the effective index between the symmetric and antisymmetric mode, leading to a shorter critical coupling length (Fig. S5D). Here, we choose a gap between the NWG 1 (NWG 2) and the MWG to be 500 nm (200 nm). In this case, $L_{cc1} = L_{cc2} = 60\ \mu$m. Further, we perform the mode propagation simulation to verify that the parallel waveguide coupler works (Fig. S5E-G). When we input light from NWG 1, it will be coupled to the TE_2 mode in the MWG (Fig. S5E, H). When we input light from the NWG 2, it will be coupled to the TE_1 mode (Fig. S5F, I). Besides, no light will be coupled to the NWG1 or NWG 2 when the TE_0 mode propagates in the MWG (Fig. S5G, J).

3 Theoretical analysis of the cascaded-mode interferometer with two modes and multiple TMCs for generating narrow linewidth spectra

In this section, we investigate the influence of the number of the TMCs N on the output spectra. We consider the case of two modes (mode 1 and mode 2). The relationship between the amplitudes of the two modes in the input and output of the cascaded-mode interferometer should be

$$\begin{pmatrix} a_{1,\text{out}} \\ a_{2,\text{out}} \end{pmatrix} = \left(\mathbf{T}_c\mathbf{T}_{\text{wg}}\right)^{N-1}\mathbf{T}_c \begin{pmatrix} a_{1,\text{in}} \\ a_{2,\text{in}} \end{pmatrix} \tag{S6}$$

Supposing that we input mode 1 into this cascaded-mode interferometer, that is, $a_{1,\text{in}} = 1$ and $a_{2,\text{in}} = 0$, we can calculate the amplitude of the output mode 2 using Eq. S6 as

$$\begin{aligned} a_{2,\text{out}} = &\frac{i\sin\left(sL_c\right)}{2^N\sqrt{\left(e^{i\beta_1 L_{\text{gap}}}+e^{i\beta_2 L_{\text{gap}}}\right)^2\cos^2\left(sL_c\right)-4e^{i(\beta_1+\beta_2)L_{\text{gap}}}}}\times \\ &\left(\left(\left(e^{i\beta_1 L_{\text{gap}}}+e^{i\beta_2 L_{\text{gap}}}\right)\cos\left(sL_c\right)+\sqrt{\left(e^{i\beta_1 L_{\text{gap}}}+e^{i\beta_2 L_{\text{gap}}}\right)^2\cos^2\left(sL_c\right)-4e^{i(\beta_1+\beta_2)L_{\text{gap}}}}\right)^N\right. \\ &\left.-\left(\left(e^{i\beta_1 L_{\text{gap}}}+e^{i\beta_2 L_{\text{gap}}}\right)\cos\left(sL_c\right)-\sqrt{\left(e^{i\beta_1 L_{\text{gap}}}+e^{i\beta_2 L_{\text{gap}}}\right)^2\cos^2\left(sL_c\right)-4e^{i(\beta_1+\beta_2)L_{\text{gap}}}}\right)^N\right) \end{aligned} \tag{S7}$$

where β_1 and β_2 are the propagation constants of the two modes. L_c is the length of the gratings (or the transmissive mode converters). L_{gap} is the gap distance between the neighboring transmissive mode converters. $s = \sqrt{\kappa^2+\delta^2}$, where $\delta = \frac{1}{2}\left(\beta_1-\beta_2-\frac{2\pi}{\Lambda}\right)$ is the phase mismatch. κ is the coupling coefficient. Λ is the grating period. The amplitude of the output mode 1 can be calculated using energy conservation: $\left|a_{1,\text{out}}\right|^2 = 1-\left|a_{2,\text{out}}\right|^2$.

When the phase matching is satisfied ($\delta = 0$) and $\kappa L_c = 0.5\pi/N$ or $\pi\pm0.5\pi/N$, or $2\pi\pm 0.5\pi/N$, and so on, we find that Eq. S7 can be further simplified to a sum of a series as

$$a_{2,\text{out}} = ie^{i\beta_2 L}\sum_{n=0}^{N-1} c_n e^{i\Delta\beta n L_{\text{gap}}} \tag{S8}$$

where $\Delta\beta = \beta_1-\beta_2$ and the coefficients c_n are listed in Table S1. The sum of this series results in narrow-linewidth spectra, with the linewidth decreasing when increasing N. Interestingly, this

characteristic is similar to the Dirichlet kernel (*57*), which is also a sum of a series, and its linewidth is inversely proportional to the number of series. However, while the coefficients of the series in the Dirichlet kernel are all the same, these coefficients in Eq. S8 are not all the same and change with N.

Table S1: **Coefficient c_n in Eq. S8.** These coefficients are calculated using Eq. S7.

$N = 2$	c_0	c_1					
	0.5	0.5					
$N = 3$	c_0	c_1	c_2				
	0.375	0.25	0.375				
$N = 4$	c_0	c_1	c_2	c_3			
	0.30177	0.19822	0.19822	0.30177			
$N = 5$	c_0	c_1	c_2	c_3	c_4		
	0.25281	0.17274	0.14887	0.17274	0.25281		
$N = 6$	c_0	c_1	c_2	c_3	c_4	c_5	
	0.21762	0.15512	0.12724	0.12724	0.15512	0.21762	
$N = 7$	c_0	c_1	c_2	c_3	c_4	c_5	c_6
	0.19107	0.14130	0.11455	0.10612	0.11455	0.14130	0.19107
...	...	...	...	...	...	...	...

4 Examining power spectral features in cascaded-mode interferometers: doughnut- and fork-shaped structures

In this section, we investigate the doughnut-shaped and fork-shaped structures in the power spectral map of cascaded-mode interferometers, shown in Fig. 2B. We re-write Eq. 5 as follows:

$$a_{1,\text{out}} = c_1 e^{-i\frac{2\pi}{\lambda} n_{\text{eff},1} L_{\text{gap}}} + c_2 e^{-i\frac{2\pi}{\lambda} n_{\text{eff},2} L_{\text{gap}}} \tag{S9}$$

with

$$c_1 = \left(\cos(sL_c) - i\frac{\delta}{s}\sin(sL_c)\right)^2 = R_1 e^{i\phi_0}$$
$$R_1 = \cos^2(sL_c) + \frac{\delta^2}{s^2}\sin^2(sL_c)$$
$$\phi_0 = -2\arctan\left(\frac{\delta}{s}\tan(sL_c)\right)$$

and

$$c_2 = -\frac{\kappa^2}{s^2}\sin^2(sL_c) = R_2 e^{i\pi}$$
$$R_2 = \frac{\kappa^2}{s^2}\sin^2(sL_c)$$

Therefore, Equation S9 can be further simplified to be

$$a_{1,\text{out}} = R_1 e^{-i\phi_1} + R_2 e^{-i\phi_2} \tag{S10}$$

with

$$\phi_1 = \frac{2\pi}{\lambda} n_{\text{eff},1} L_{\text{gap}} - \phi_0$$
$$\phi_2 = \frac{2\pi}{\lambda} n_{\text{eff},2} L_{\text{gap}} - \pi$$
$$R_1 + R_2 = 1$$

Then, we calculate the converted power of the TE_2 mode as

$$
\begin{aligned}
|a_{2,\mathrm{out}}|^2 &= 1 - |a_{1,\mathrm{out}}|^2 \\
&= 1 - |R_1 e^{-i\phi_1} + R_2 e^{-i\phi_2}|^2 \\
&= 1 - \left(R_1^2 + R_2^2 + 2R_1R_2\cos(\phi_1 - \phi_2)\right) \\
&= 1 - \left((R_1 + R_2)^2 - 2R_1R_2 + 2R_1R_2\cos(\phi_1 - \phi_2)\right) \\
&= 1 - (1 - 2R_1R_2 + 2R_1R_2\cos(\phi_1 - \phi_2)) \\
&= 2R_1R_2(1 - \cos(\phi_1 - \phi_2)) \\
&= 2R_1R_2(1 + \cos(\phi_1 - \phi_2 - \pi)) \\
&= 2\eta(1 - \eta)(1 + \cos(\Delta\phi))
\end{aligned}
\tag{S11}
$$

with

$$
\Delta\phi = \phi_1 - \phi_2 - \pi = \frac{2\pi}{\lambda}(n_{\mathrm{eff},1} - n_{\mathrm{eff},2})L_{\mathrm{gap}} + 2\arctan(\frac{\delta}{s}\tan(sL_c))
$$

$$
\eta = R_2 = \frac{\kappa^2}{s^2}\sin^2(sL_c)
$$

By the way, we can also obtain Eq. S11 directly from the amplitude of TE_2 mode, $a_{2,\mathrm{out}}$, which can be calculated using Equation 4 as :

$$
\begin{aligned}
a_{2,\mathrm{out}} = &-i\frac{\kappa}{s}\sin(sL_c)\left(\cos(sL_c) - i\frac{\delta}{s}\sin(sL_c)\right)e^{-i\frac{2\pi}{\lambda}n_{\mathrm{eff},1}L_{\mathrm{gap}}} \\
&-i\frac{\kappa}{s}\sin(sL_c)\left(\cos(sL_c) + i\frac{\delta}{s}\sin(sL_c)\right)e^{-i\frac{2\pi}{\lambda}n_{\mathrm{eff},2}L_{\mathrm{gap}}}
\end{aligned}
\tag{S12}
$$

Now, using the factors provided in Eq. S11, we compute the power spectral map and systematically analyze the contributions of individual factors, as illustrated in Fig. S24. This analysis shows that the power spectral maps calculated using Eq. 5 and Eq. S11 are identical, as demonstrated in Figs. S24A and S24F. This equivalence confirms that the two methods, one based on Eq. 5 and the other on Eq. S11, are consistent in their implementation and results.

Doughnut-Shaped Structures

Let us begin with the factor η in Eq. S11. The parameter η consists of two components, $\frac{\kappa^2}{s^2}$ and $\sin^2(sL_c)$. The spectral map calculated with the factor $\sin^2(sL_c)$ exhibits periodic structures in

the radial direction, as shown in Fig. S24H. This periodicity arises because $s = \sqrt{\kappa^2 + \delta^2}$, where sweeping κ (on the y-axis) modifies the coupling strength κL_c, while sweeping the wavelength (on the x-axis) alters the phase mismatch δ. The phase mismatch is approximated by: $\delta \approx \frac{\pi \Delta n_{\text{eff}}(\lambda-\lambda_0)}{\lambda_0^2}$. Meanwhile, the spectral map calculated with the factor $\frac{\kappa^2}{s^2}$ reveals that the bandwidth increases with the coupling strength κL_c, as depicted in Fig. S24G. The bandwidth can be determined from: $\mathrm{I}(\lambda) = \frac{\kappa^2}{s^2} = \frac{\kappa^2}{\kappa^2+\delta^2(\lambda)} = \kappa^2 / \left(\kappa^2 + (\frac{\pi \Delta n_{\text{eff}}(\lambda-\lambda_0)}{\lambda_0^2})^2\right)$. At the center wavelength λ_0, where phase matching is satisfied, $\mathrm{I}(\lambda_0) = 1$. At λ_1, where $\mathrm{I}(\lambda_1) = 0.5$, the relationship becomes: $\lambda_1 - \lambda_0 = \frac{\kappa \lambda_0^2}{\pi \Delta n_{\text{eff}}}$. The bandwidth is therefore given by:

$$\Delta\lambda_{\text{BW}} = 2(\lambda_1 - \lambda_0) = \frac{2\kappa\lambda_0^2}{\pi \Delta n_{\text{eff}}} \tag{S13}$$

This equation explicitly shows that the bandwidth increases with the coupling coefficient κ. When combined, the two factors $\frac{\kappa^2}{s^2}$ and $\sin^2(sL_c)$ produce the pattern observed in Fig. S24C. The spectral map calculated with $1-\eta$ yields a pattern that is the inverse of η, as seen in Fig. S24B. Consequently, the term $2\eta(1-\eta)$ generates the doughnut-shaped structures depicted in Fig. S24D.

Fork-Shaped Structures

Next, we analyze the factor $1 - \cos\Delta\phi$ in Eq. S11. The phase difference $\Delta\phi$ has two components:

(I) $\frac{2\pi}{\lambda}(n_{\text{eff},1} - n_{\text{eff},2})L_{\text{gap}}$, which arises from the phase difference between the two modes as they propagate in the multimode waveguide section of the cascaded-mode interferometer.

(II) $2\arctan\left(\frac{\delta}{s}\tan(sL_c)\right)$, which is introduced by the mode converters.

The first component produces spectral interference patterns, while the second contributes a π-phase shift under specific conditions: when the wavelength corresponds to the center wavelength (where $\delta = 0$), and simultaneously, the coupling strength satisfies $\kappa L_c = \frac{\pi}{2} + n\pi$ $(n = 0, 1, 2, \ldots)$. This combination results in the fork-shaped structures observed in Fig. S24E at these positions.

Fig. S1-24

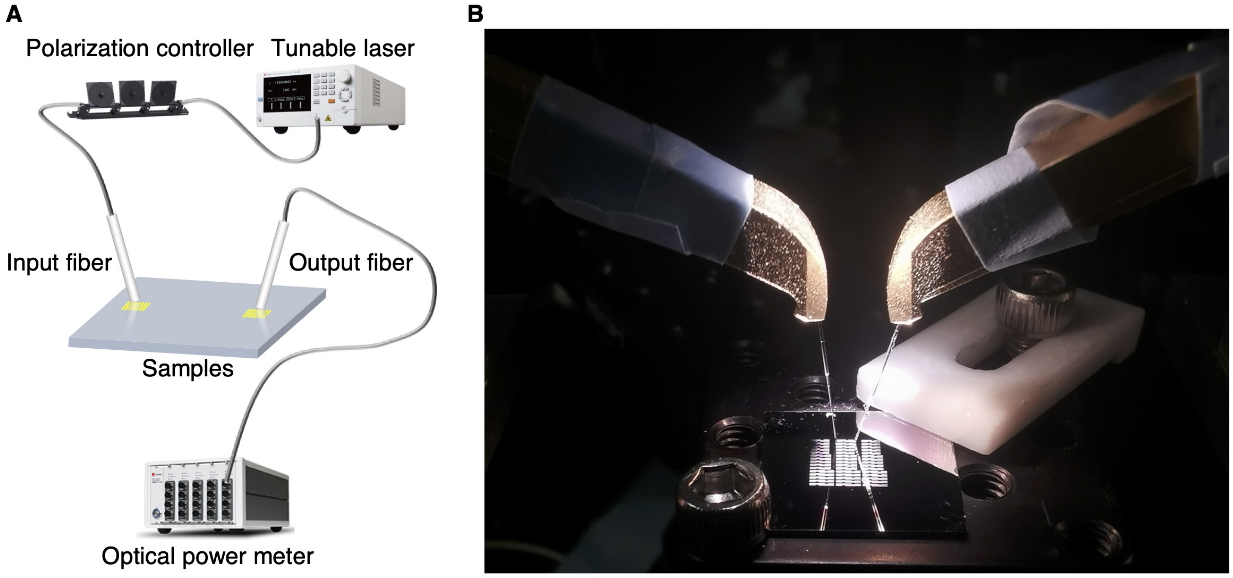

Figure S1: **Measurement setup.** (**A**) Schematic of the measurement setup. (**B**) Optical image of the two-fiber-probe system.

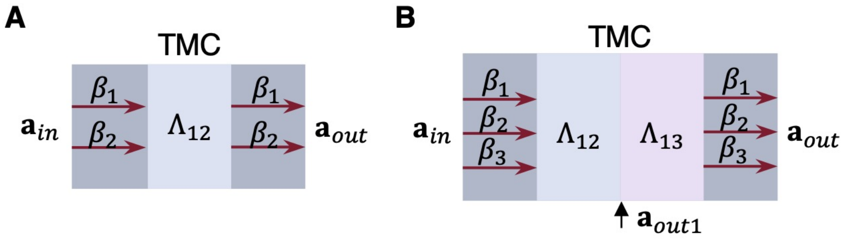

Figure S2: **Transmissive mode converters (TMC) for two modes (A)and three modes (B).**

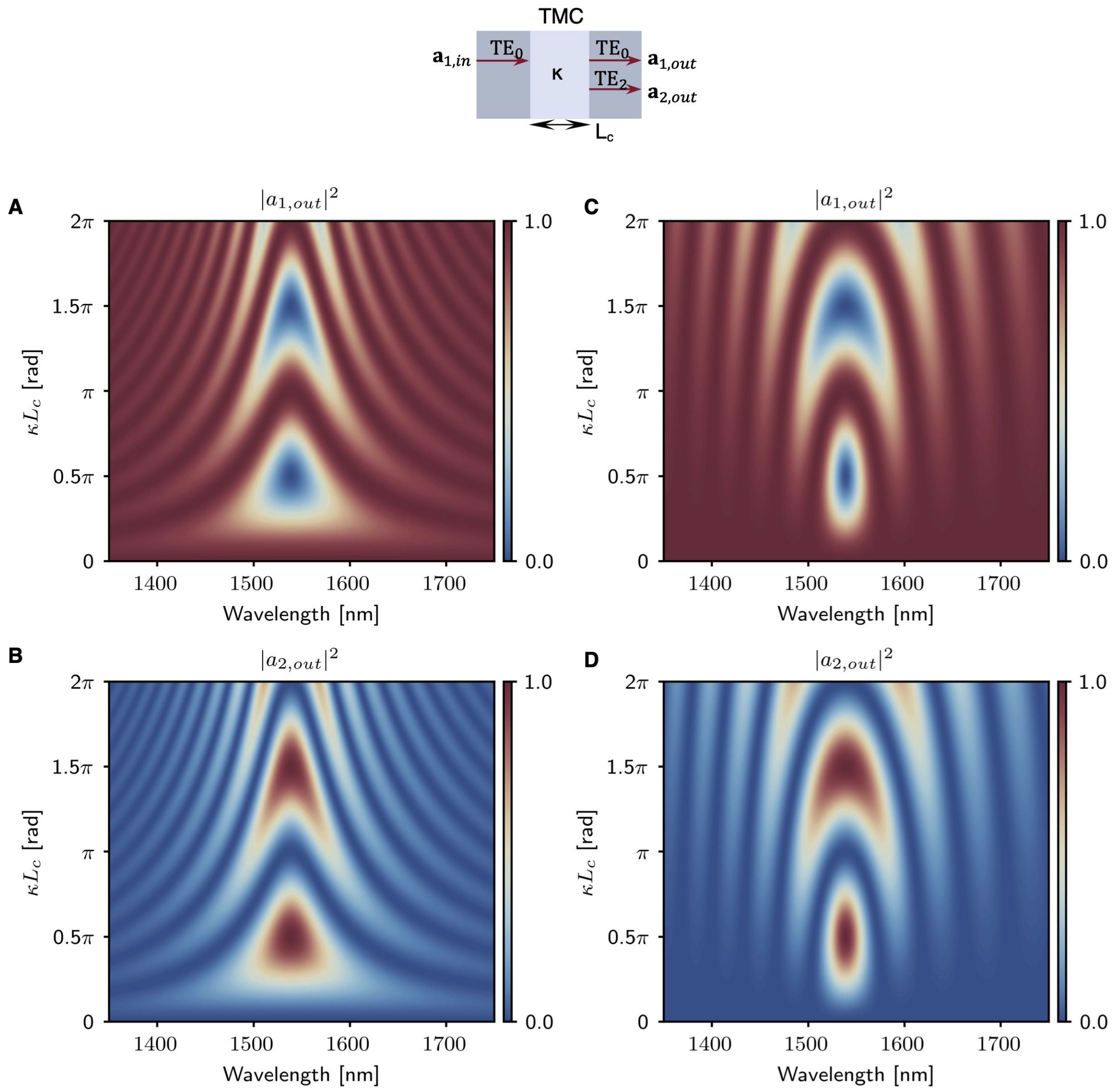

Figure S3: **Theoretical calculations of the grating-assisted mode conversion in a multimode silicon waveguide with** TE$_0$ **mode input.** The output power $\left(|a_{\text{out}}|^2\right)$ of the TE$_0$ (**A**, **C**) and TE$_2$ (**B**, **D**) mode when varying the grating length L_c and keeping the coupling coefficient κ constant $\left(\pi/66.65\ \mu\text{m}^{-1}\right)$ (**A**, **B**), and when varying the coupling coefficient κ and keeping the grating length L_c constant (66.65 μm) (**C**, **D**).

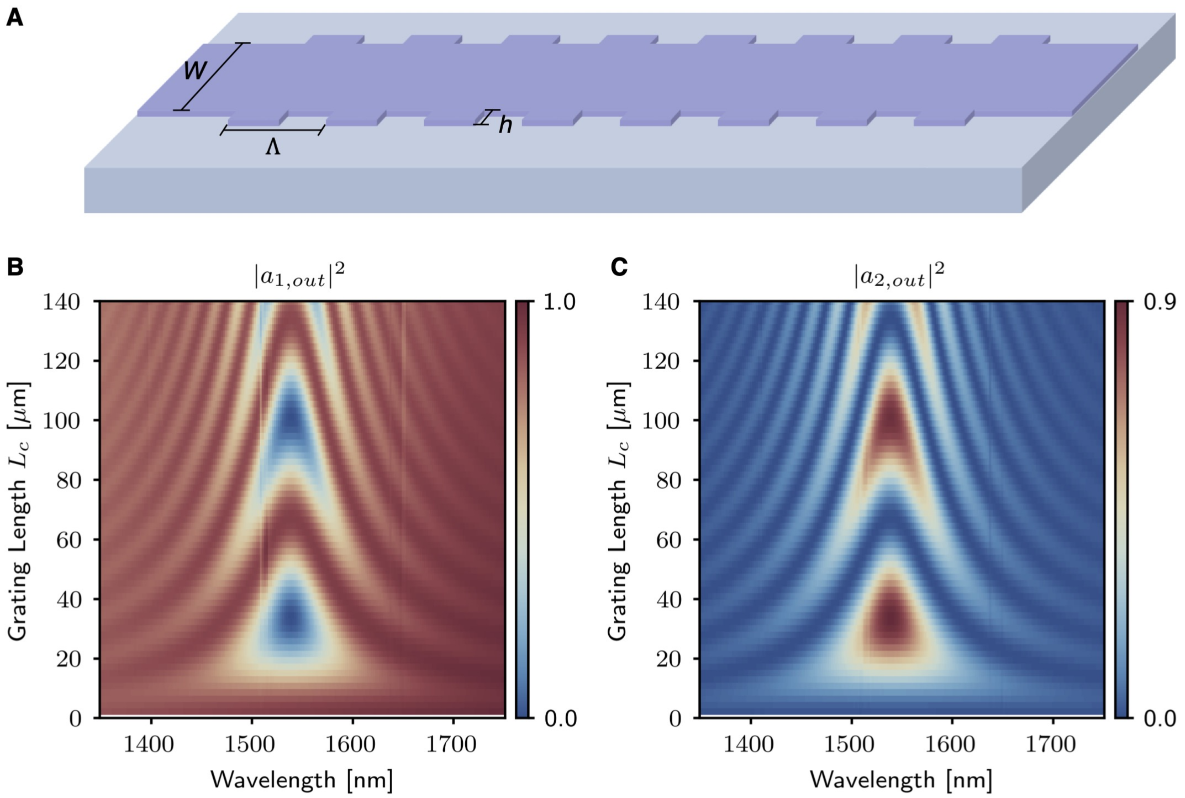

Figure S4: **Simulation results of the grating-assisted mode conversion in a multimode silicon waveguide with** TE_0 **mode input.** (**A**) Schematic of the grating-assisted transmissive mode converter. The output power of the TE_0 (**B**) and TE_2 (**C**) modes when varying the grating length L_c. Waveguide width: W = 1100 nm, grating period: Λ = 2150 nm, grating depth: h = 40 nm.

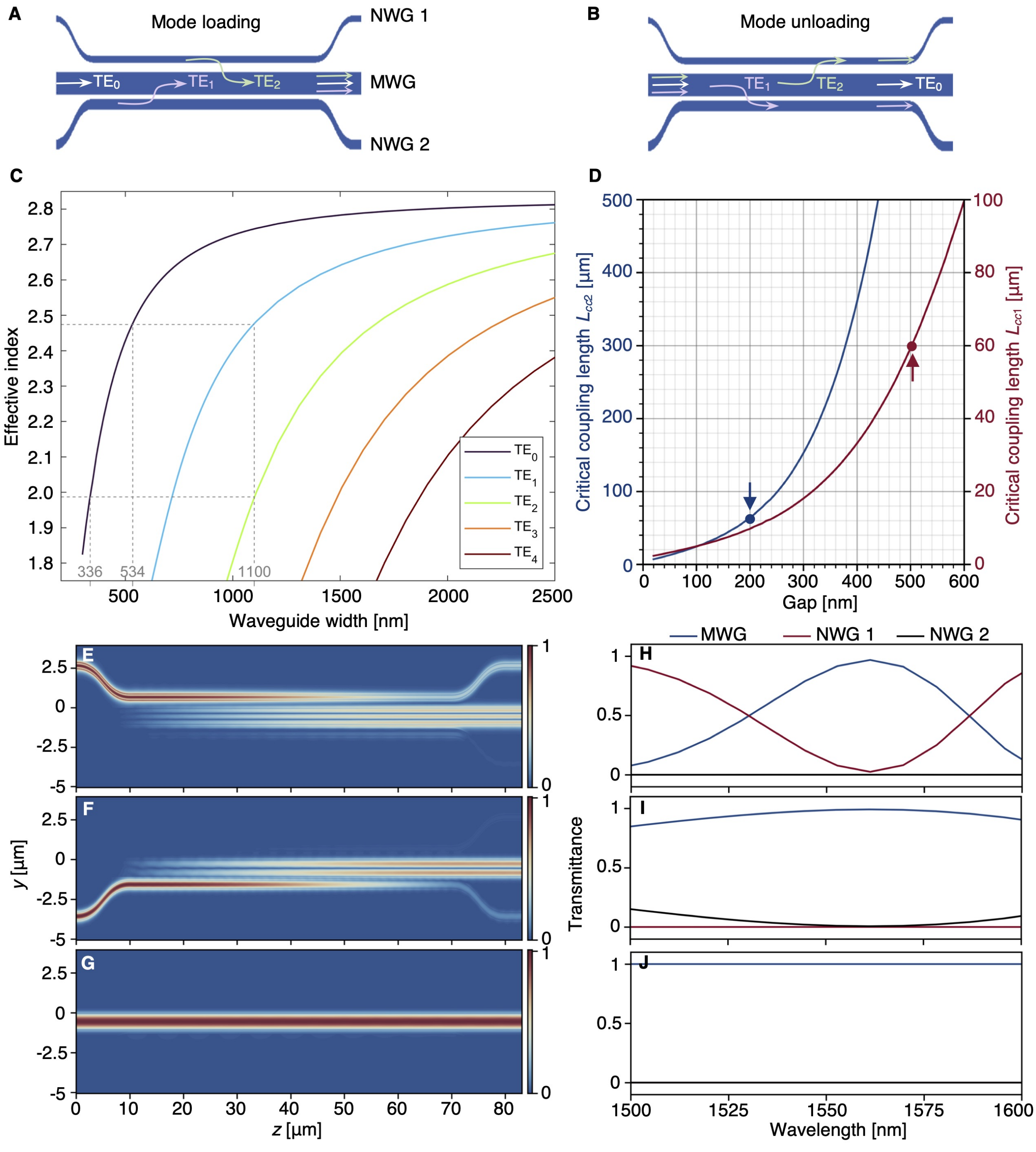

Figure S5: **(Caption next page.)**

Figure S5: **(Previous page.) Parallel waveguide couplers for mode loading and unloading.** Schematic of the mode loading (**A**) and unloading (**B**) in a multimode waveguide using parallel waveguide couplers. Three waveguides are placed together, where the middle waveguide is a multimode waveguide (MWG) with a width of $W = 1100$ nm. The nanowaveguide 1 (NWG 1) is used to load and unload the TE_2 mode in the MWG. The nanowaveguide 2 (NWG 2) is used to load and unload the TE_1 mode in the MWG. (**C**) Effective indexes of the multiple transverse electric modes of a single waveguide vary with its width. The phase matching condition is satisfied when the width of the NWG 1 and 2 are 336 nm and 534 nm, respectively. (**D**) Critical coupling lengths for NWG 1 (L_{cc1}) and NWG 2 (L_{cc2}) vary with the gap between the NWG 1 (NWG 2) and the MWG. (**E-G**) Simulated electric field distributions when loading TE_2 (**E**), TE_1 (**F**), and TE_0 (**G**) modes using the NWG 1, NWG 2, and MWG at the wavelength of 1550 nm. The coupling lengths for the NWG1 and NWG 2 are both 60 μm. (**H-J**) Transmittance spectra for the NWG 1 (red), NWG 2 (black), and MWG (blue) when a fundamental mode (TE_0) inputs from the NWG 1 (**H**), NWG 2 (**I**), and MWG (**J**).

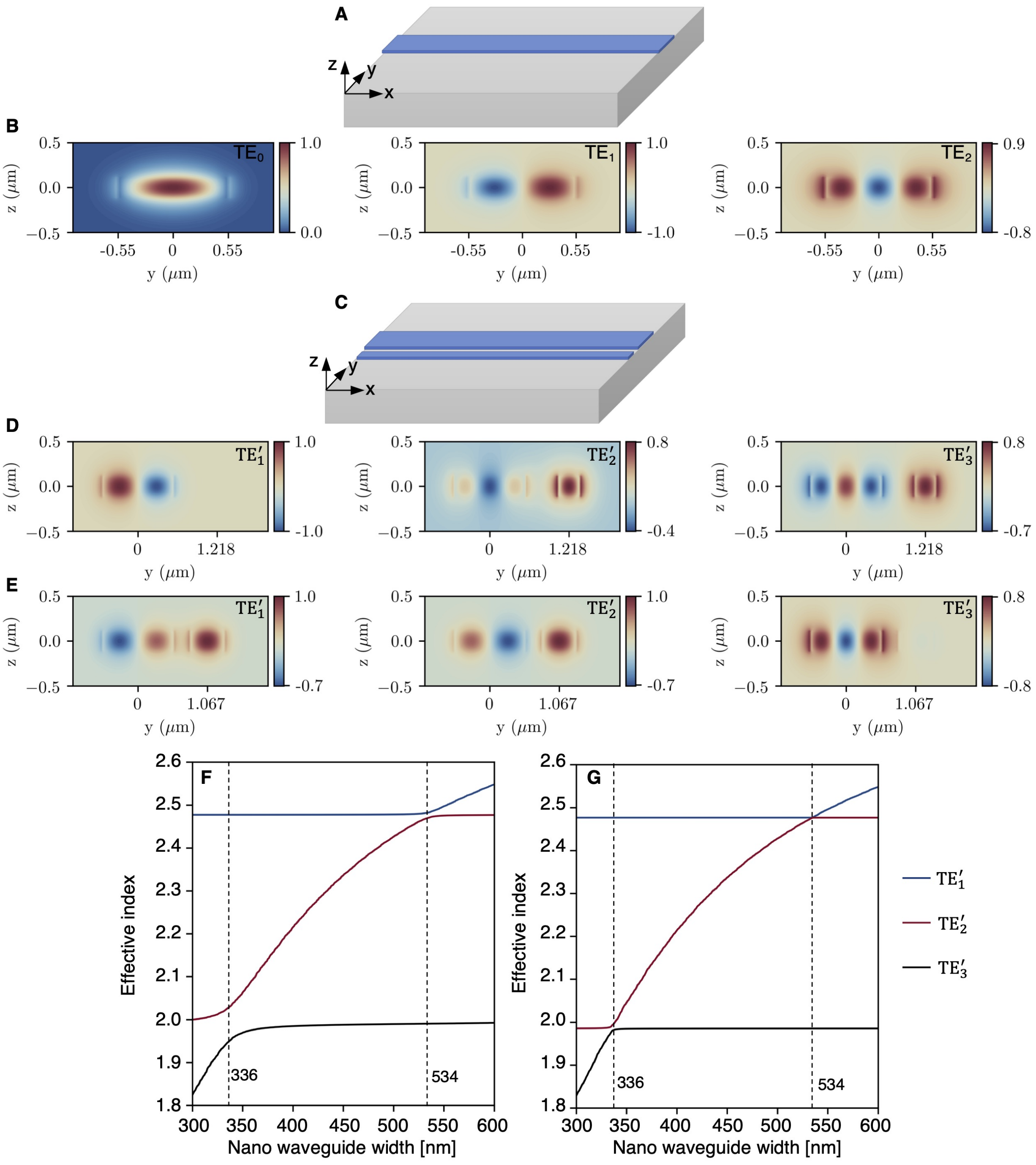

Figure S6: **(Caption next page.)**

Figure S6: **(Previous page.) Eigenmodes of the parallel waveguides.** Schematic of a multimode waveguide (**A**) and the parallel waveguides composed of a multimode waveguide and a nanowaveguide (**C**). (**B**) Mode profiles $\left(\mathrm{Re}\left(E_y\right)\right)$ of the eigenmode $\mathrm{TE}_0, \mathrm{TE}_1$, and TE_2 of the multimode waveguide with the width of $W = 1100$ nm. (**D, E**) Mode profiles of the eigenmode $\mathrm{TE}_1, \mathrm{TE}_2$, and TE_3 of the parallel waveguides. The width of the multimode waveguide is 1100 nm. The width of the nano waveguide is 336 nm in (**D**) and 534 nm in (**E**). (**F, G**) The effective indexes of the eigenmode $\mathrm{TE}_1, \mathrm{TE}_2$, and TE_3 of the parallel waveguides vary with the width of the nano waveguide. The gap between the multimode waveguide and the nano waveguide is 500 nm in (**D, G**) and 200 nm in (**E, F**).

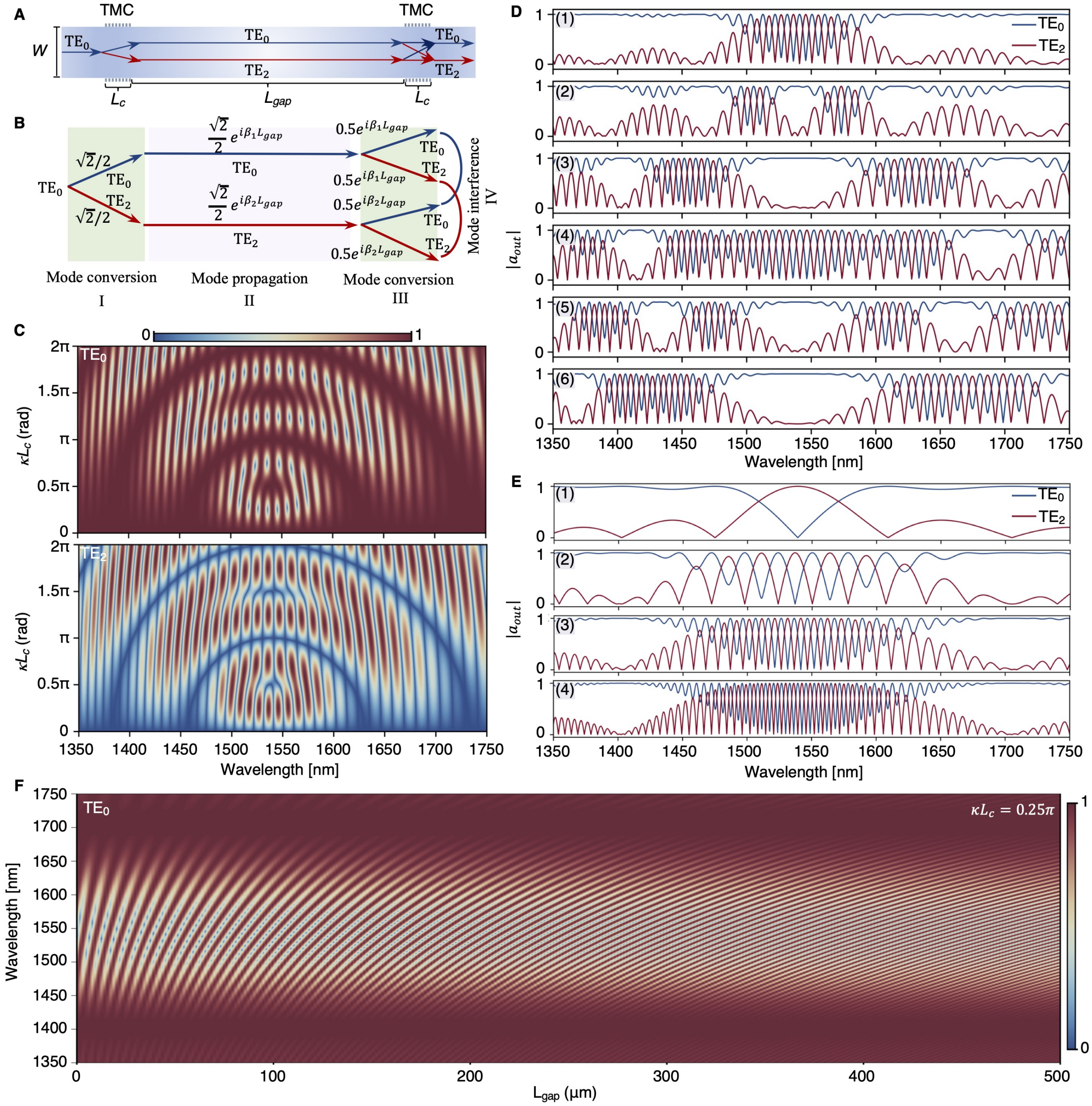

Figure S7: **(Caption next page.)**

Figure S7: **(Previous page.) Theoretical calculations of the most simple cascaded-mode interferometer.** (**A**) Schematic of an on-chip cascaded-mode interferometer. Two transverse electric modes (TE_0 and TE_2) in the multimode waveguide are used. The cascaded-mode interferometer includes two transmissive mode converters (TMCs) with a gap L_{gap}. (**B**) Working principle of the cascaded-mode interferometer. The TMCs function as 50:50 power splitters, meaning the power converting efficiency of the TMCs is 50%. Four processes happen sequentially in the cascade mode interferometer: (I) the input TE_0 mode is split into the TE_0 and TE_2 modes, each with the amplitudes of $\frac{\sqrt{2}}{2}$; (II) the TE_0 and TE_2 modes propagate a distance L_{gap} and obtain propagation phases of $e^{-i\beta_1 L_{\text{gap}}}$ and $e^{-i\beta_2 L_{\text{gap}}}$, respectively. (II) the TE_2 modes, now with different phases, are split into TE_0 and TE_2 mode again; (IV) the two components of the TE_0 or TE_2 mode, each with different phases, interfere with each other. The phase change during the mode conversion is ignored in (**B**). (**C**) Amplitude spectra ($|a_{\text{out}}(\lambda)|$) of the TE_0 and TE_2 modes in the output of the cascaded-mode interferometer varies with the coupling strength κL_c. We sweep the coupling coefficient κ and fix the coupling length $L_c = 30\ \mu$m. The grating period is $\Lambda = 2126$ nm. $L_{\text{gap}} = 232.5\ \mu$m. (**D**) Cut lines at some coupling strengths in (**C**). (**D**-1) $\kappa L_c = 0.25\pi$, (**D**-2) $\kappa L_c = 0.5\pi$, (**D**-3) $\kappa L_c = \pi$, (**D**-4) $\kappa L_c = 1.25\pi$, (**D**-5) $\kappa L_c = 1.5\pi$, (**D**-6) $\kappa L_c = 2\pi$. (**E**) Amplitude spectra ($|a_{\text{out}}(\lambda)|$) of the output TE_0 and TE_2 modes for different L_{gap} when $\kappa L_c = 0.25\pi$. (**E**-1) $L_{\text{gap}} = 0$, (**E**-2) $L_{\text{gap}} = 50\ \mu$m, (**E**-3) $L_{\text{gap}} = 150\ \mu$m, (**E**-4) $L_{\text{gap}} = 250\ \mu$m. (**F**) Amplitude spectra ($|a_{\text{out}}(\lambda)|$) of the output TE_0 modes vary with L_{gap}.

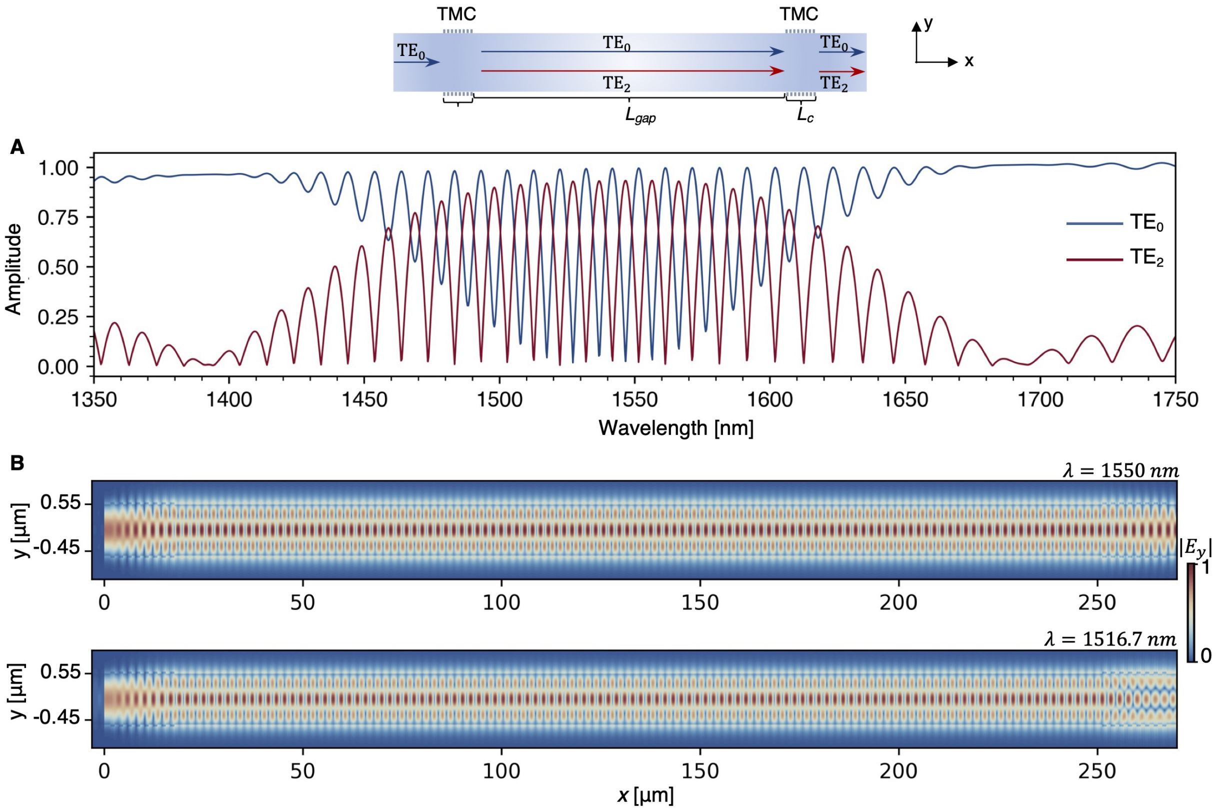

Figure S8: **Simulation results of the most simple cascaded-mode interferometer.** (**A**) Amplitude spectra ($|a_{\text{out}}(\lambda)|$) of the TE_0 and TE_2 modes at the output of the cascaded-mode interferometer. (**B**) Simulated electric field ($|E_y|$) distributions in the cascaded-mode interferometer at the peak wavelengths 1550 nm and 1516.7 nm of the TE_0 and TE_2 modes, respectively. Parameters used in simulations: silicon waveguide width $W = 1100$ nm, silicon waveguide thickness $t = 220$ nm, $L_{\text{gap}} = 232.5\ \mu$m, grating period $\Lambda = 2126$ nm, grating depth $h = 40$ nm, grating number m = 8, grating length $L_c = \text{m}\Lambda = 17\ \mu$m, coupling coefficient: $\kappa = 0.25\pi/L_c = 0.046\ \mu\text{m}^{-1}$.

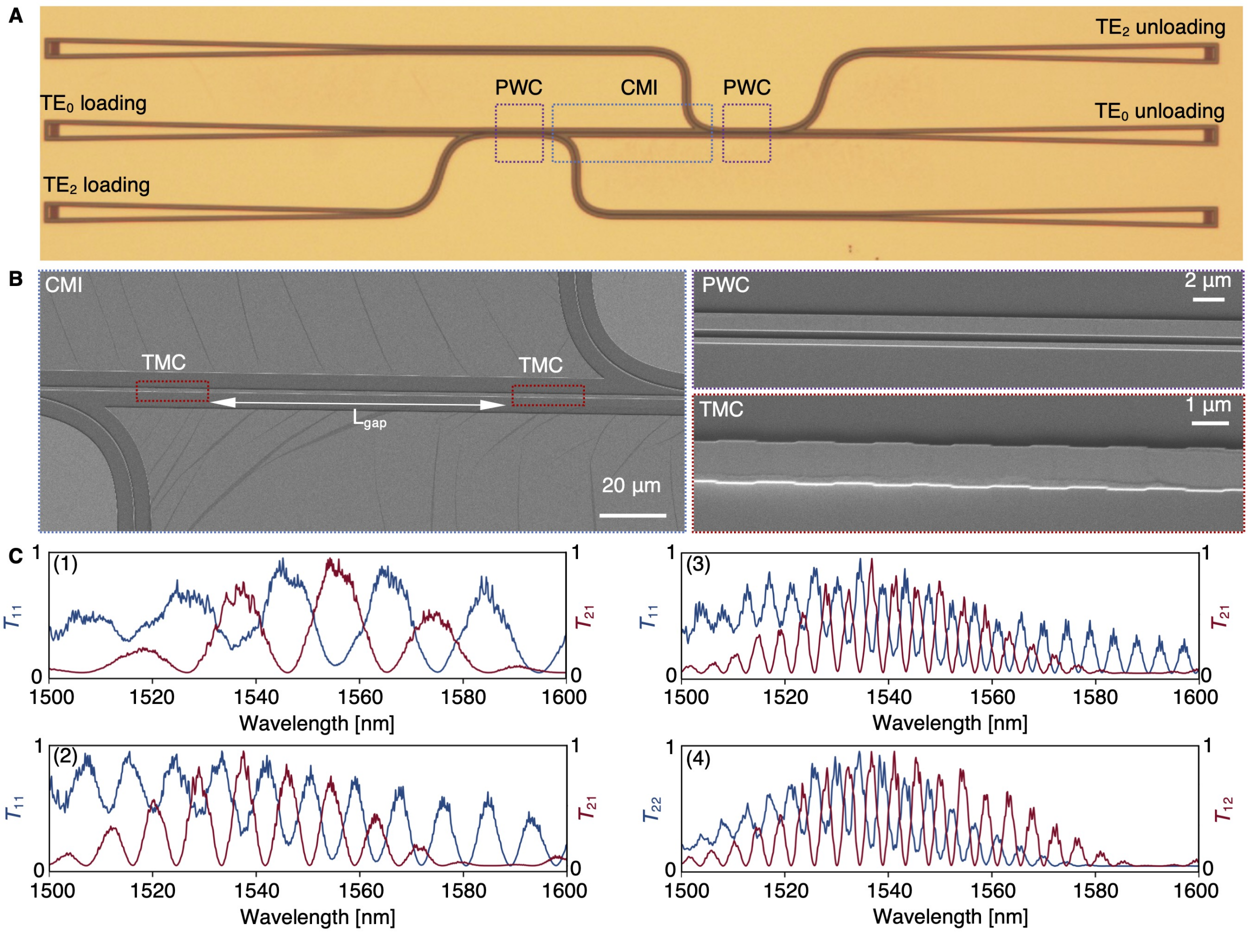

Figure S9: **Experiment results of the most simple cascaded-mode interferometer.** (**A**) Optical microscope image of a fabricated on-chip cascaded-mode interferometer (CMI). (**B**) Scanning electron microscope images of the cascaded-mode interferometer, parallel waveguide coupler (PWC), and transmissive mode converter (TMC). (**C**) Measured interference spectra at the output of the cascaded-mode interferometer. T_{ij}: normalized power of the output mode i for the case that the mode j inputs (i and j take 1 and 2). Mode 1: TE_0, mode 2: TE_2. (**C**-1) L_{gap} = 100 μm, (**C**-2) L_{gap} = 250 μm, (**C**-3) L_{gap} = 500 μm, (**C**-4) L_{gap} = 500 μm. The measured free spectral ranges when L_{gap} = 100 μm, 250 μm, and 500 μm are 19.4 nm, 8.5 nm, and 4.4 nm, respectively, which agree well with the theoretical calculations (21.5 nm, 8.6 nm, and 4.3 nm, respectively) using the formula $\Delta\lambda_{FSR} = \frac{\lambda^2}{(n_{g1}-n_{g2})L_{gap}}$, where n_{g1} = 4.85 and n_{g2} = 3.75 are the group indexes of the TE_0 and TE_2 modes, respectively.

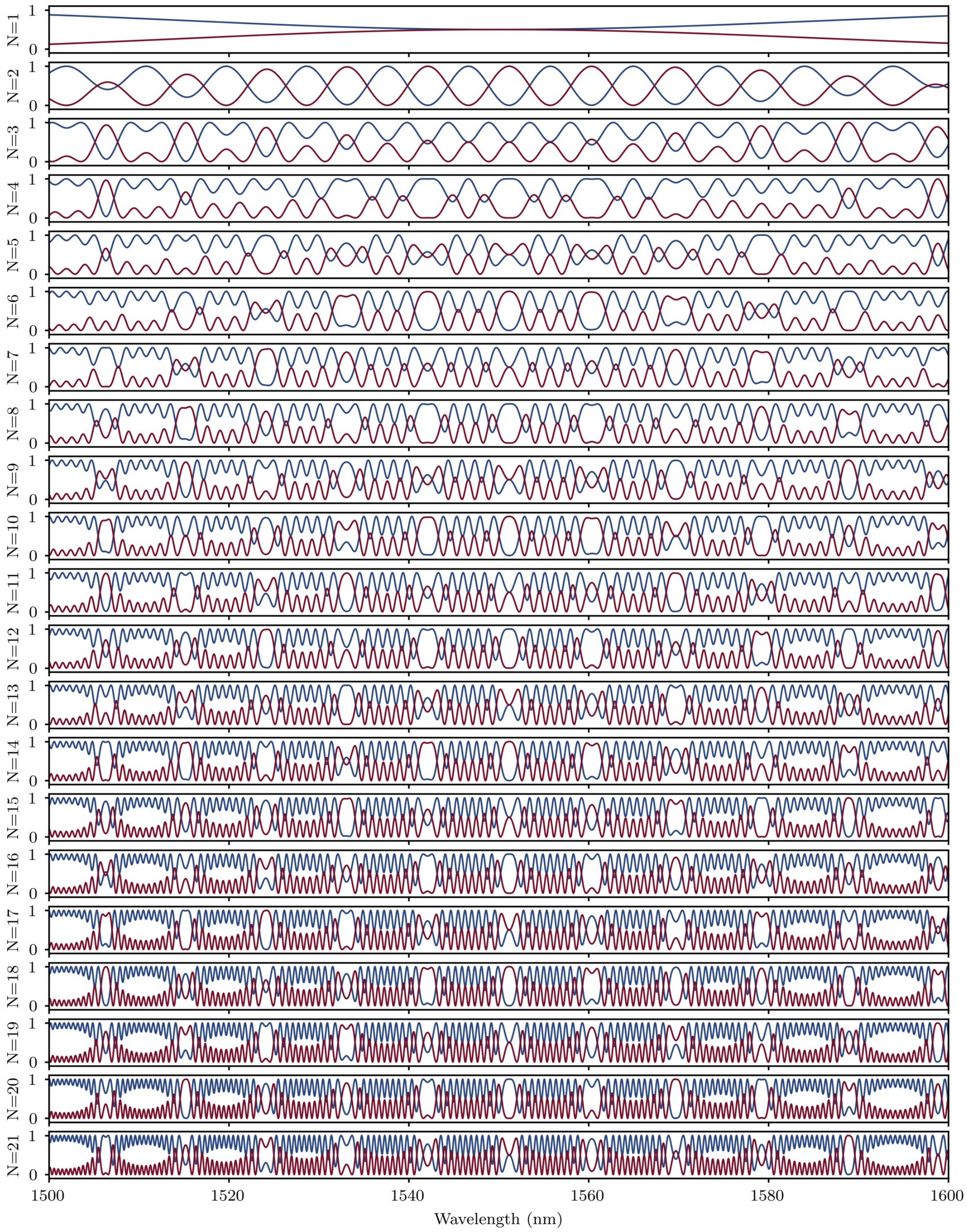

Figure S10: **Line plots of Fig. 3B.** Calculated output power spectra ($|a_{\text{out}}|^2$) of the TE_0 (blue curves) and TE_2 (red curves) mode with varying the number of TMCs when $\kappa L_c = 0.25\pi$.

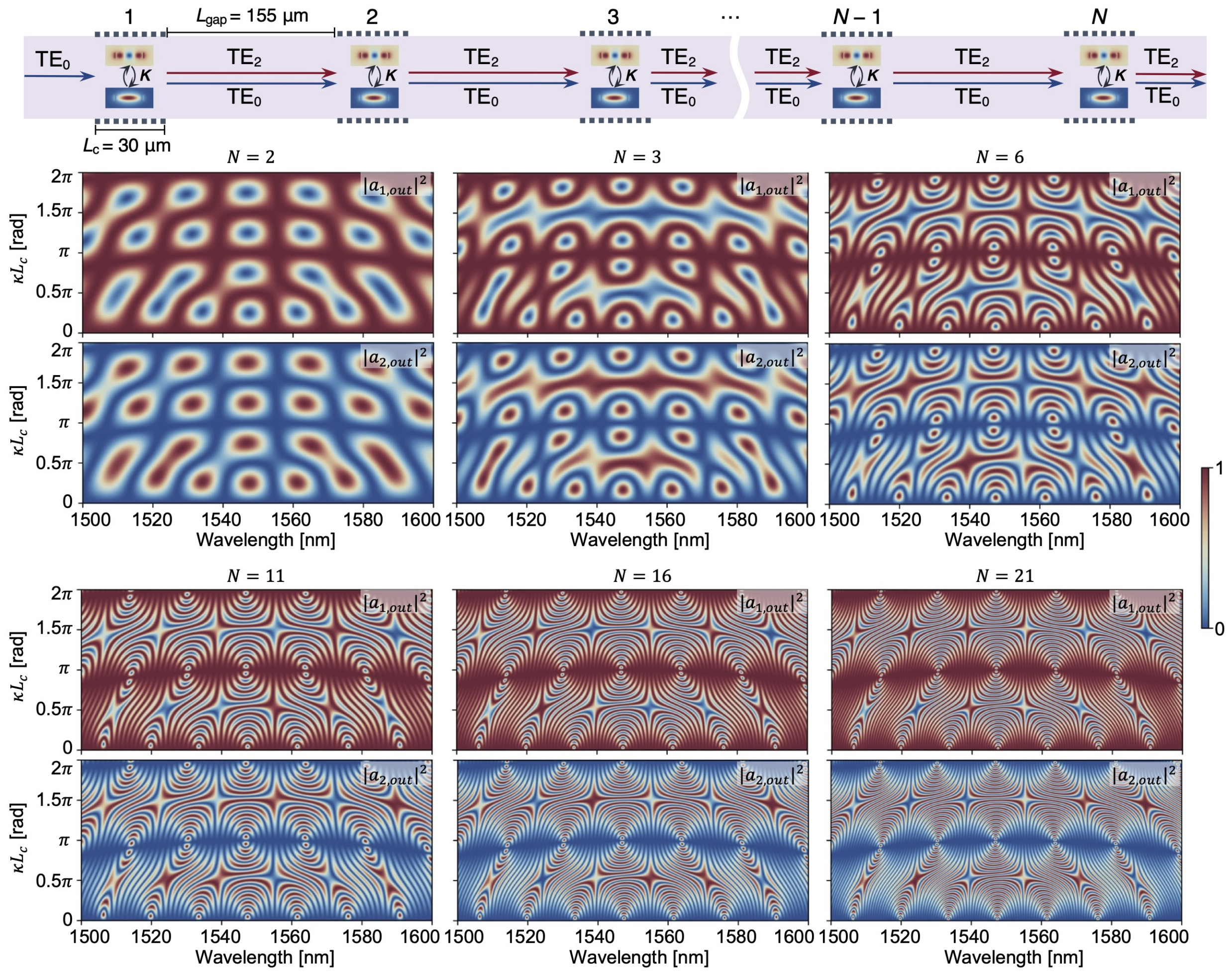

Figure S11: **Theoretical calculations of the output power spectra** $\left(|a_{\mathbf{out}}|^2\right)$ **of the cascaded-mode interferometer with multiple TMCs when** N **= 2, 3, 6, 11, 16, and 21.** We vary the coupling strength κ_c. The TE_0 and TE_2 modes are used. All the TMC is identical, and its grating length is $L_c = 30\ \mu$m. The gap between every two neighboring TMCs remains the same as $L_{\text{gap}} = 155\ \mu$m. $|a_{1,\text{out}}|^2$ and $|a_{2,\text{out}}|^2$ represent the power of output TE_0 and TE_2 modes.

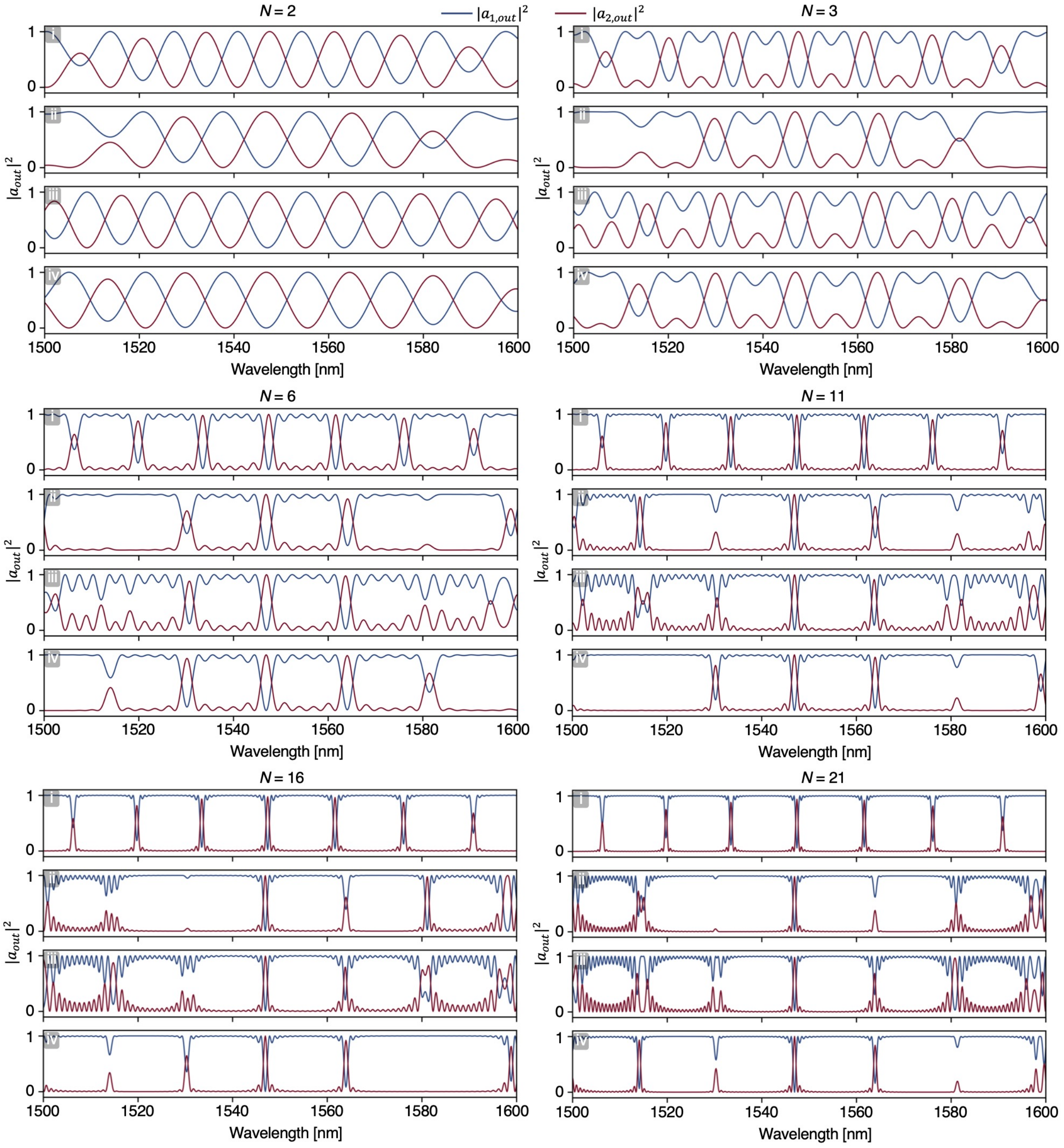

Figure S12: **Cut lines of Fig. S11.** (i) $\kappa L_c = 0.5\pi/N$. (ii) $\kappa L_c = (N - 0.5)\pi/N$. (iii) $\kappa L_c = (N + 0.5)\pi/N$. (iv) $\kappa L_c = (2N - 0.5)\pi/N$, where N = 2, 3, 6, 11, 16, and 21. $|a_{1,\text{out}}|^2$ and $|a_{2,\text{out}}|^2$ represent the power of output TE_0 and TE_2 modes.

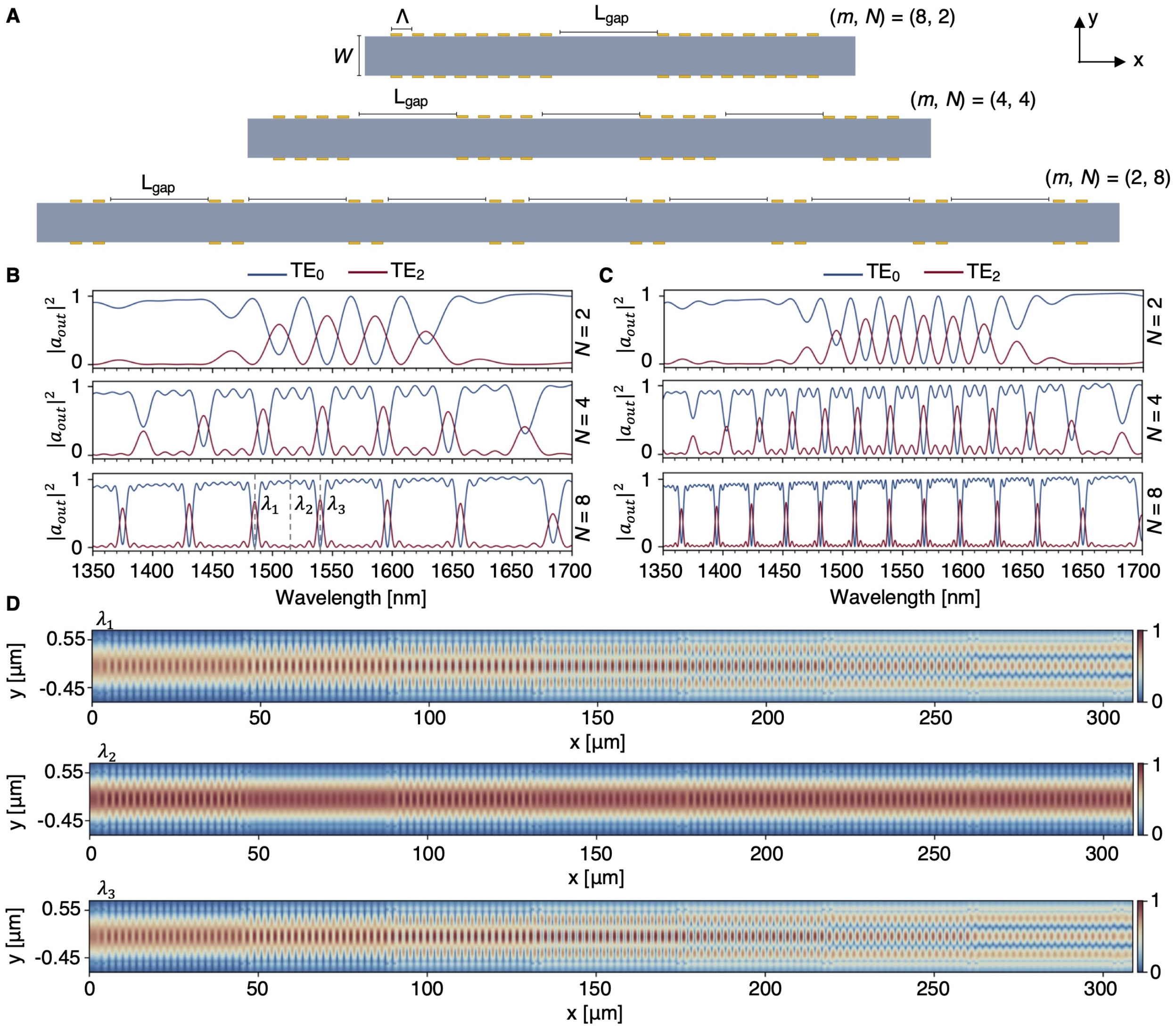

Figure S13: **Simulation results of the cascaded-mode interferometer with multiple TMCs.** (**A**) Schematic of the cascaded-mode interferometer with the number of the TMCs $N = 2$, 4, and 8 used in simulations. By adjusting the number of the grating periods with N such that m $= 16/N$, we can ensure that the coupling strength (κL_c) of the mode conversion TMC satisfies $\kappa L_c = 0.5\pi/N$. (**B, C**) Power spectra of the output TE_0 and TE_2 modes when $L_{\text{gap}} = 38.75$ μm (**A**) and $L_{\text{gap}} = 77.5$ μm (**B**). (**D**) Simulated electric field distributions at the wavelengths $\lambda_1 = 1484.76$ nm, $\lambda_2 = 1514.91$ nm, and $\lambda_3 = 1539.46$ nm, as indicated in (**A**). The input mode is TE_0 mode. Parameters used in simulations: waveguide width $W = 1100$ nm, waveguide thickness $t = 220$ nm, grating period $\Lambda = 2150$ nm, grating depth $h = 40$ nm, and coupling coefficient $\kappa = \pi/(32\Lambda)$ $= 0.046$ μm^{-1}.

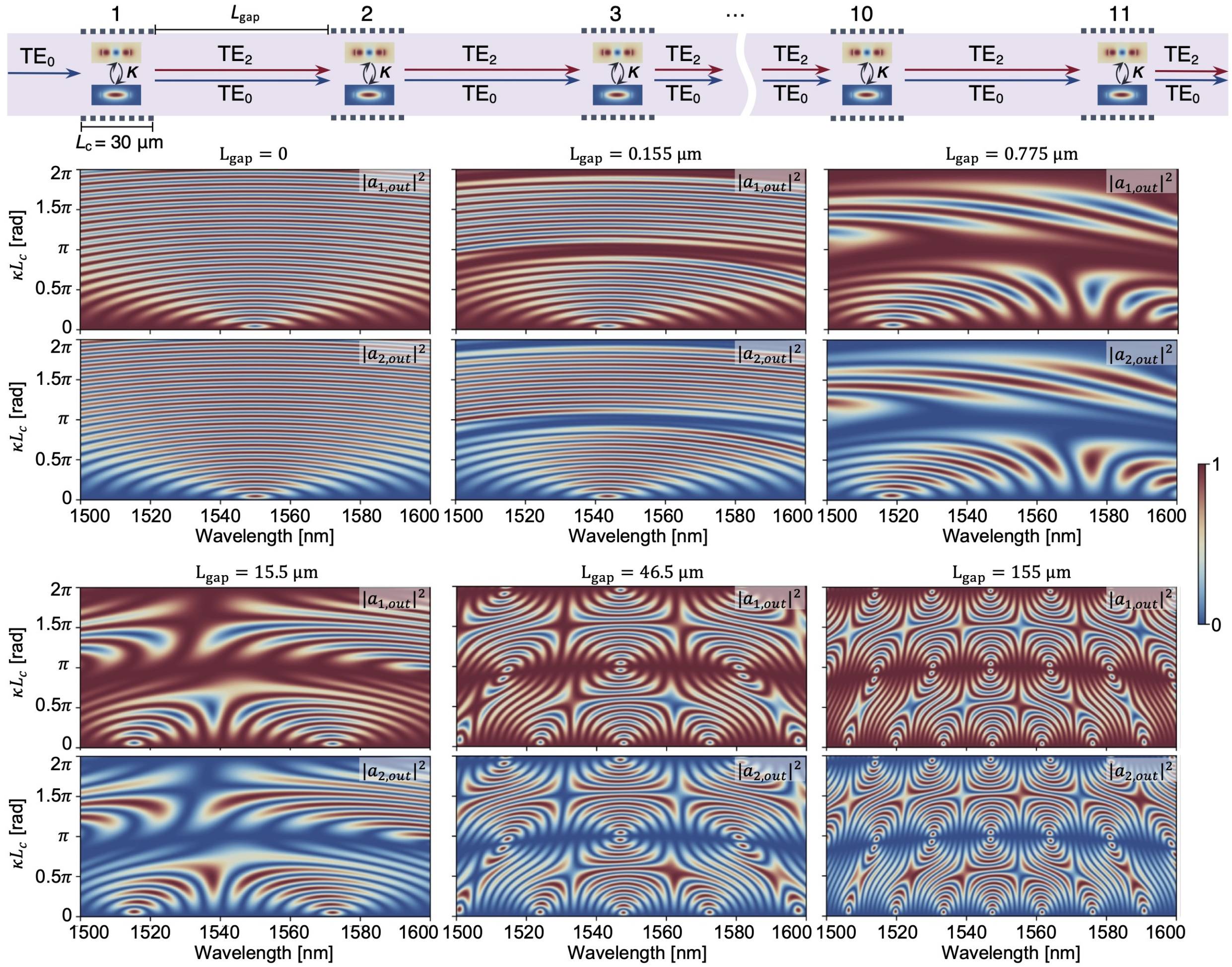

Figure S14: **Theoretical calculations of the cascaded-mode interferometer's output power spectra ($|a_{\mathbf{out}}|^2$) with multiple TMCs when $L_{\mathbf{gap}}$ = 0, 0.155, 0.775, 15.5, 46.5, and 155 μm.** We input the TE_0 mode into the cascaded-mode interferometer and vary the coupling strength κL_c. $|a_{1,\text{out}}|^2$ and $|a_{2,\text{out}}|^2$ represent the power of output TE_0 and TE_2 modes. The number of mode converters N is 11. All the TMCs are identical, and the grating length L_c is 30 μm.

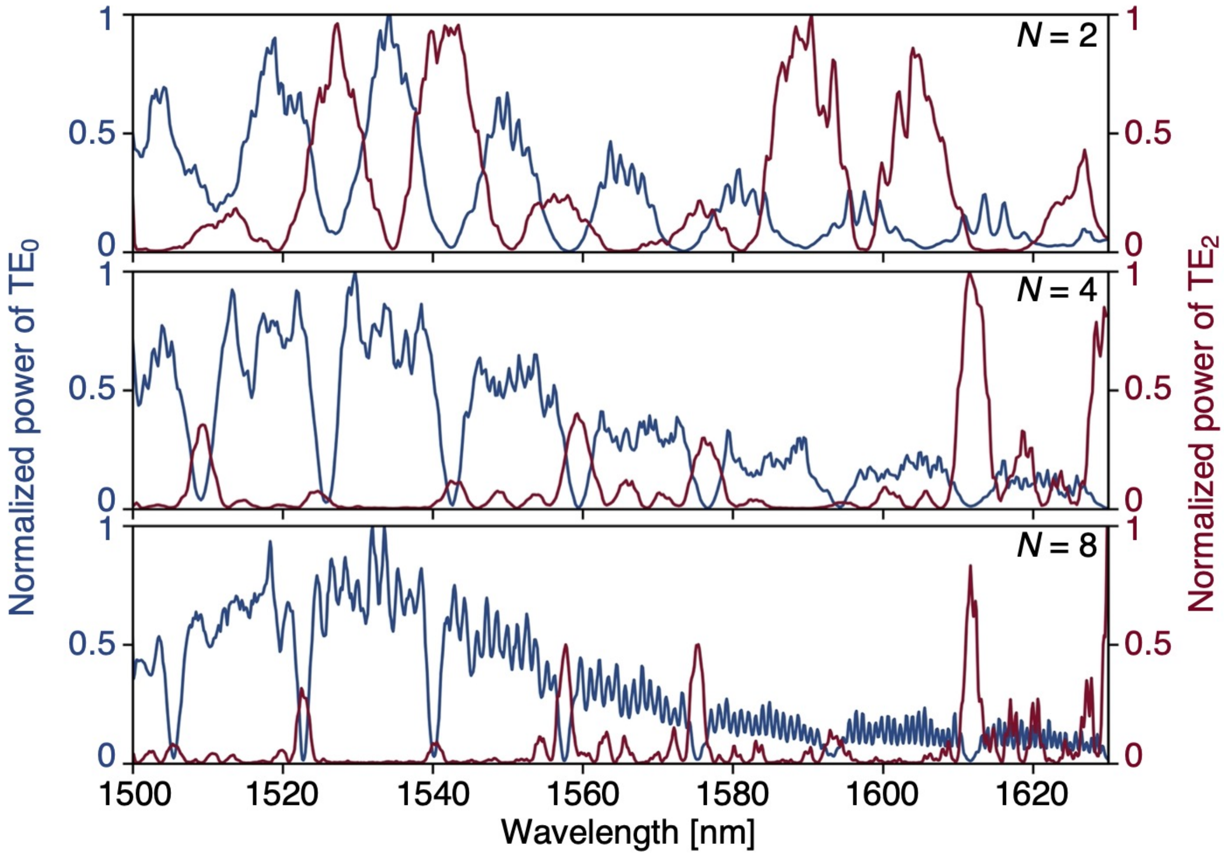

Figure S15: **The measured power spectra of the output modes** TE_0 **and** TE_2 **of the cascaded-mode interferometer with multiple TMCs when** $L_{\mathbf{gap}}$ = **125** μ**m.** Top: $N = 2$; middle: $N = 4$; bottom: $N = 8$.

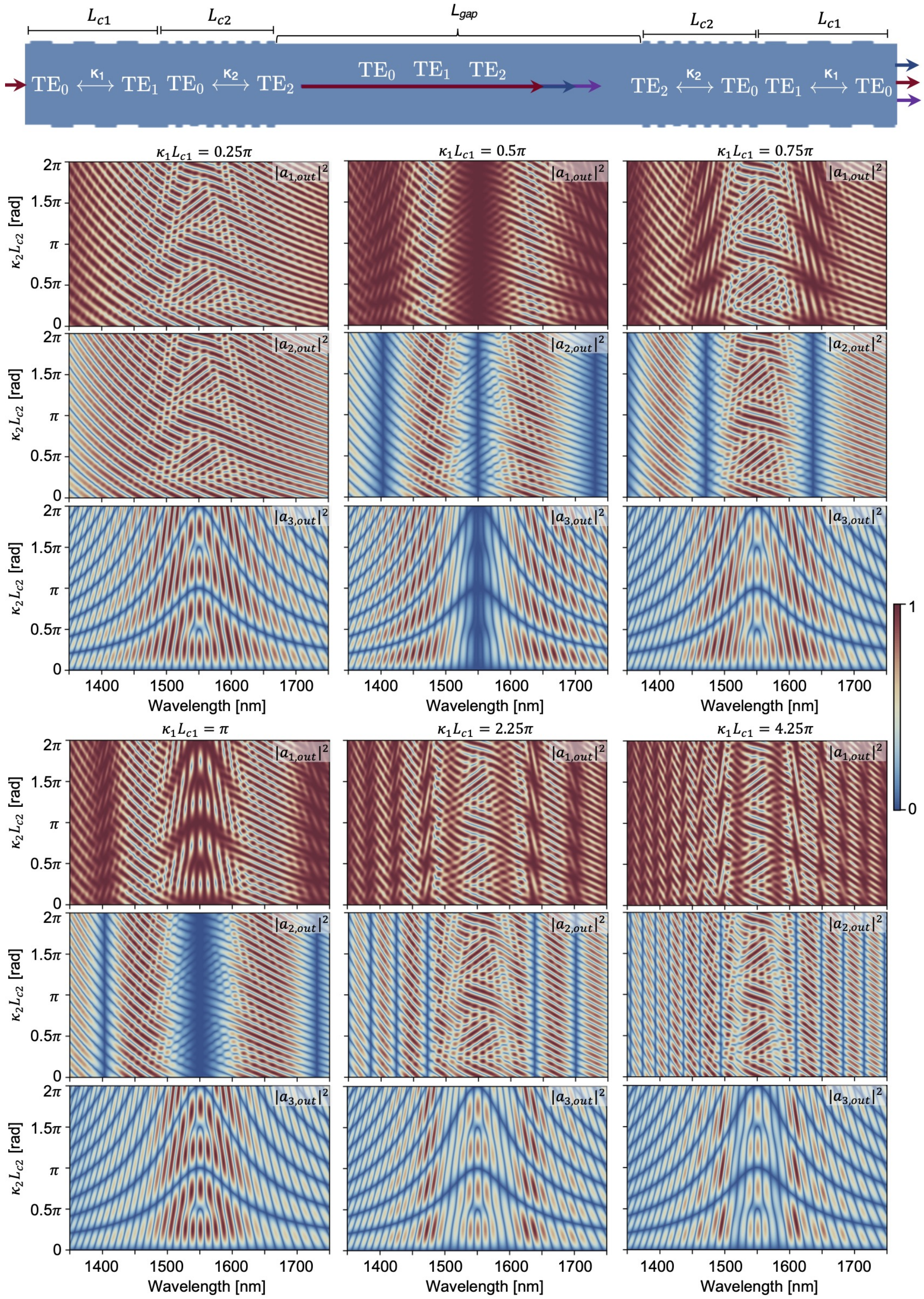

Figure S16: **(Caption next page.)**

Figure S16: **(Previous page.) Theoretical calculations of the cascaded-mode interferometer with two mode converters and three modes when varying the grating lengths.** We vary the grating lengths (L_{c1} and L_{c2}) and fix the coupling coefficients (κ_1 and κ_2). The TE_0, TE_1, and TE_2 modes are used, and their effective indexes are 2.74, 2.47, and 1.98, respectively. The coupling coefficient κ_1 of the mode coupling between TE_0 and TE_1 is 0.033 μm^{-1}. The coupling coefficient κ_2 of the mode coupling between TE_0 and TE_2 is 0.048 μm^{-1}. The gap (L_{gap}) between the transmissive mode converters is 250 μm.

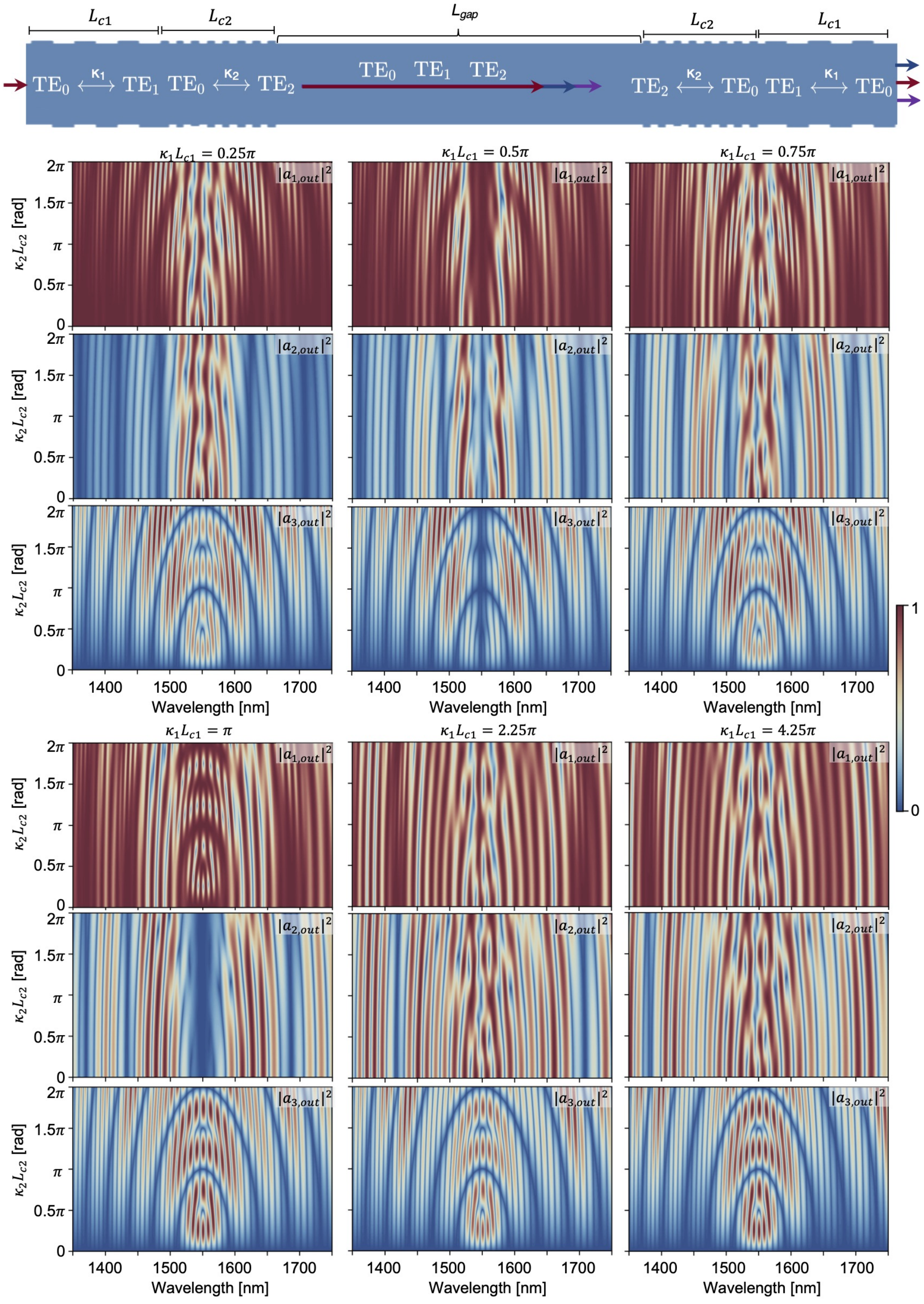

Figure S17: **(Caption next page.)**

Figure S17: **(Previous page.) Theoretical calculations of the cascaded-mode interferometer with two mode converters and three modes when varying the coupling coefficients.** We vary the coupling coefficients (κ_1 and κ_2) and fix the grating lengths (L_{c1} and L_{c2}). The TE_0, TE_1, and TE_2 modes are used, and their effective indexes are 2.74, 2.47, and 1.98, respectively. The grating lengths L_{c1} and L_{c2} are 96 μm and 64 μm, respectively. The gap (L_{gap}) between the transmissive mode converters is 250 μm.

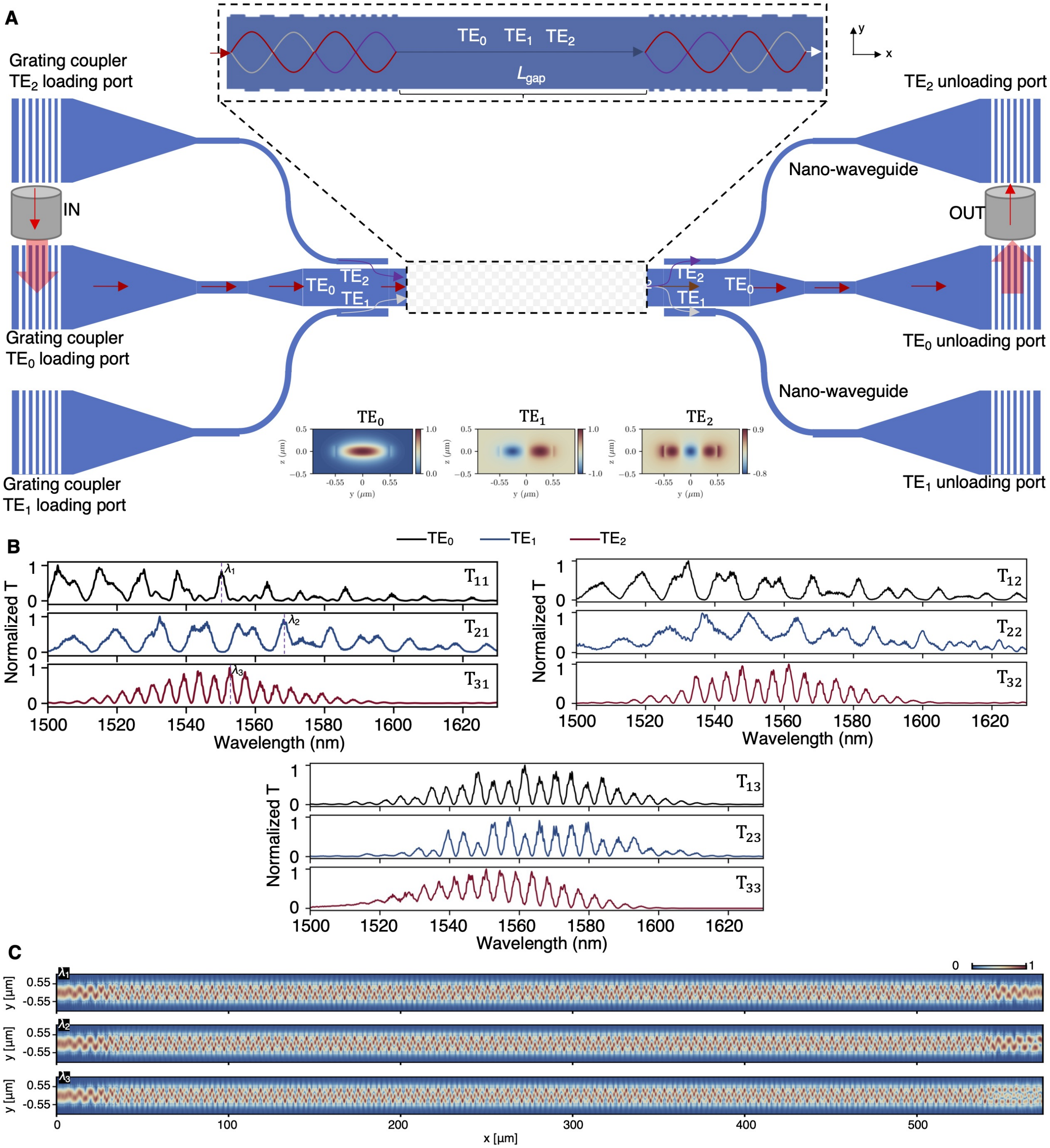

Figure S18: (Caption next page.)

Figure S18: **(Previous page.) cascaded-mode interferometer involving three modes for parallel spectra engineering.** (**A**) Schematic of a cascaded-mode interferometer involving the TE_0, TE_1, and TE_2 modes. Parallel waveguide couplers are used to load and unload these modes into and out of the cascaded-mode interferometer. L_{gap} = 500 μm. (**B**) Measured output power spectra of the TE_0, TE_1, and TE_2 modes. T_{ij} (i, j = 1, 2, and 3) in (**B**) represents the normalized power of the output mode i for the case that the mode j inputs (mode 1 : TE_0; mode 2: TE_1; mode 3: TE_2). (**C**) Simulated electric field distributions at the peak wavelength λ_1, λ_2, and λ_3 of the output TE_0, TE_1, and TE_2 mode, respectively, as indicated in (**B**).

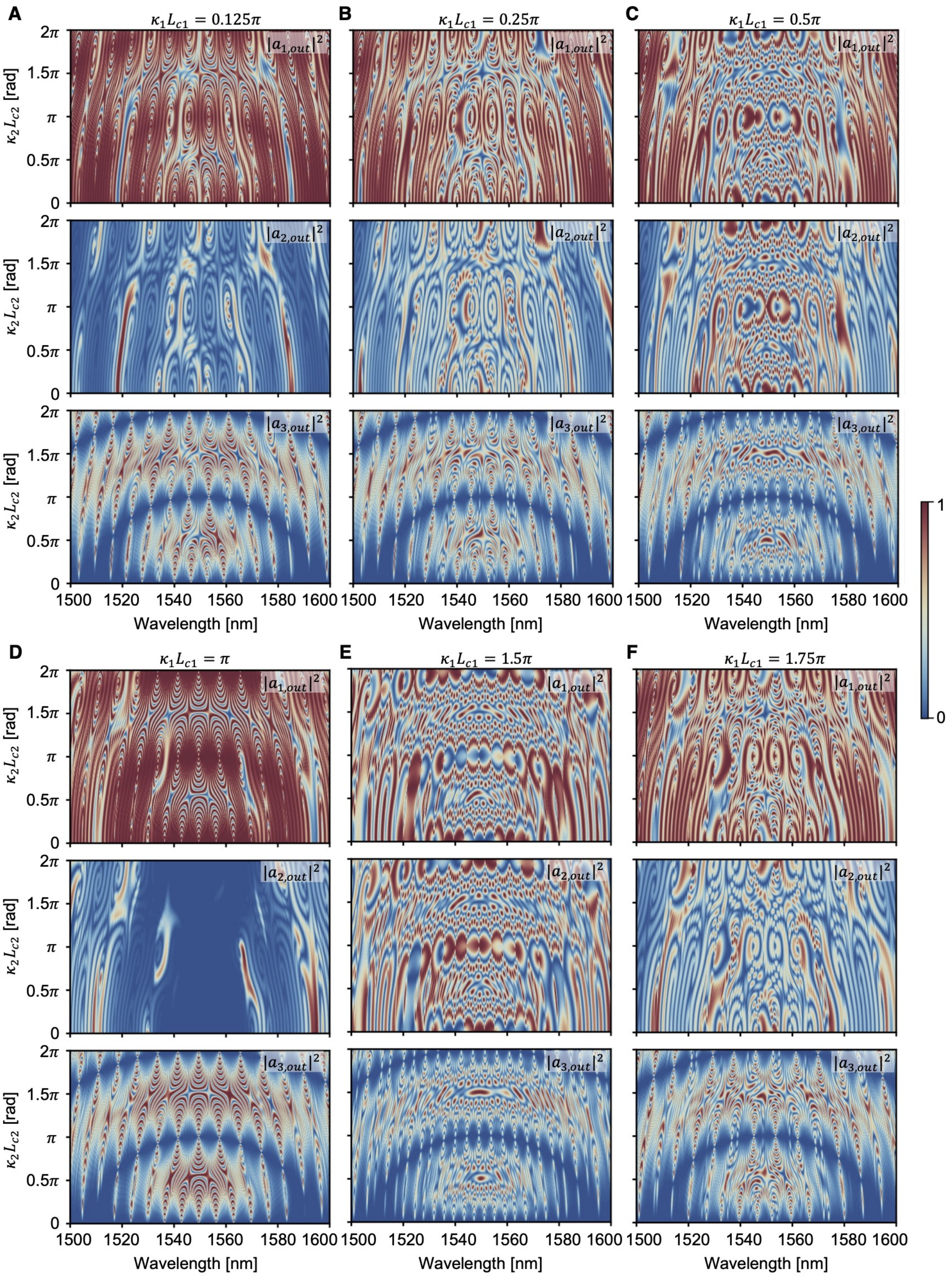

Figure S19: **(Caption next page.)**

Figure S19: **(Previous page.) Theoretical calculations of the cascaded-mode interferometer with seventeen TMCs and three modes when varying the coupling coefficients.** The TE_0, TE_1, and TE_2 modes are used, whose output spectra are noted as $|a_{1,\text{out}}|^2$, $|a_{2,\text{out}}|^2$, and $|a_{3,\text{out}}|^2$, respectively. The effective indexes $n_{\text{eff}1}$, $n_{\text{eff}2}$, and $n_{\text{eff}3}$ of these three modes are 2.74, 2.47, and 1.98, respectively. The coupling lengths L_{c1} and L_{c2} are 96 μm and 64 μm, respectively. The gap between the neighboring transmissive mode converters (L_{gap}) is 250 μm. The grating periods of TMCs for the mode conversion between the TE_0 and TE_1 and between TE_0 and TE_2 are 6023 nm and 2126 nm, respectively. The number of transmissive mode converters N is 17. We input the TE_0 mode. (**A**) $\kappa_1 L_{c1} = 0.125\pi$. (**B**) $\kappa_1 L_{c1} = 0.25\pi$. (**C**) $\kappa_1 L_{c1} = 0.5\pi$. (**D**) $\kappa_1 L_{c1} = \pi$. (**E**) $\kappa_1 L_{c1} = 1.5\pi$. (**F**) $\kappa_1 L_{c1} = 1.75\pi$.

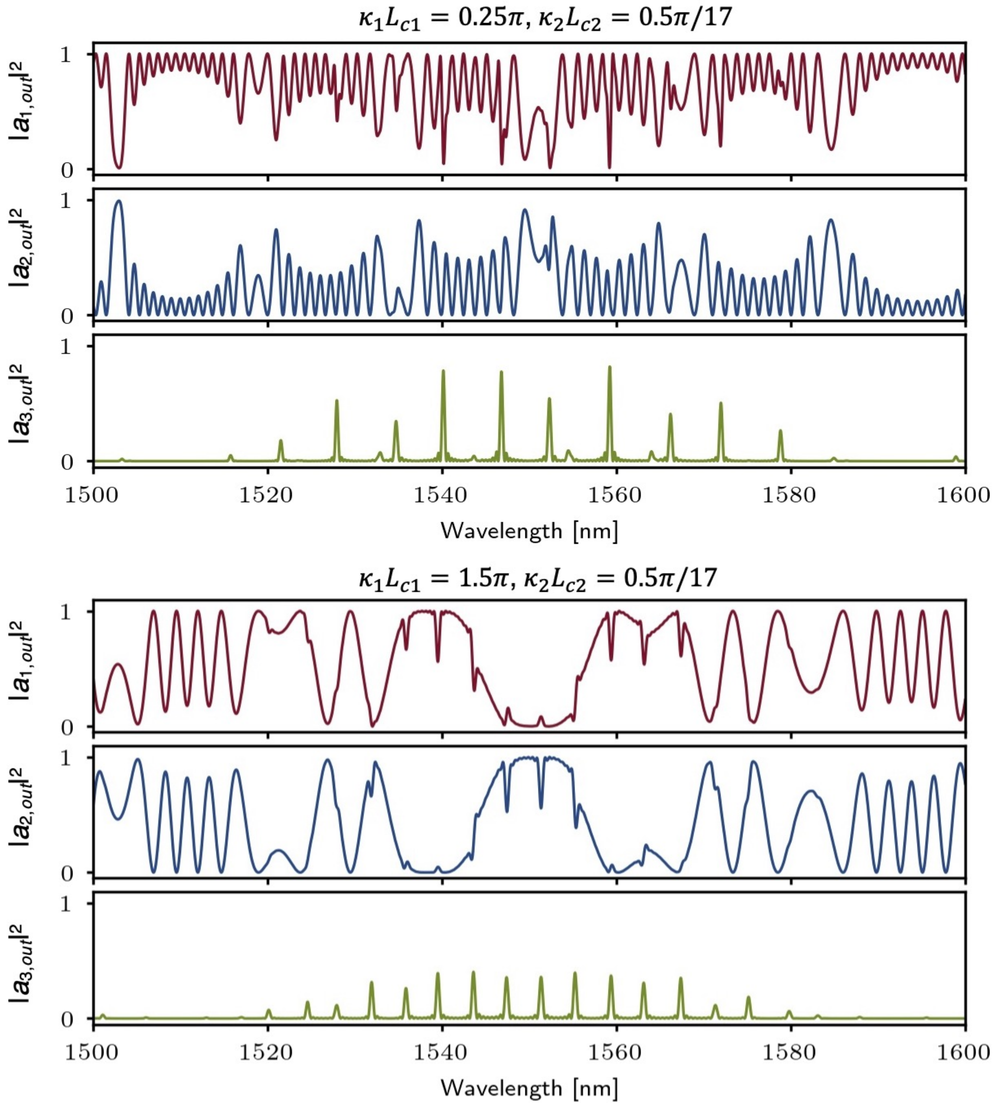

Figure S20: **Line cuts of Fig. S19 when** $\kappa_1 L_{c1} = 0.25\pi, \kappa_2 L_{c2} = 0.5\pi/17$ **(top) and when** $\kappa_1 L_{c1} = 1.5\pi, \kappa_2 L_{c2} = 0.5\pi/17$ **(bottom).** The number of transmissive mode converters N is 17.

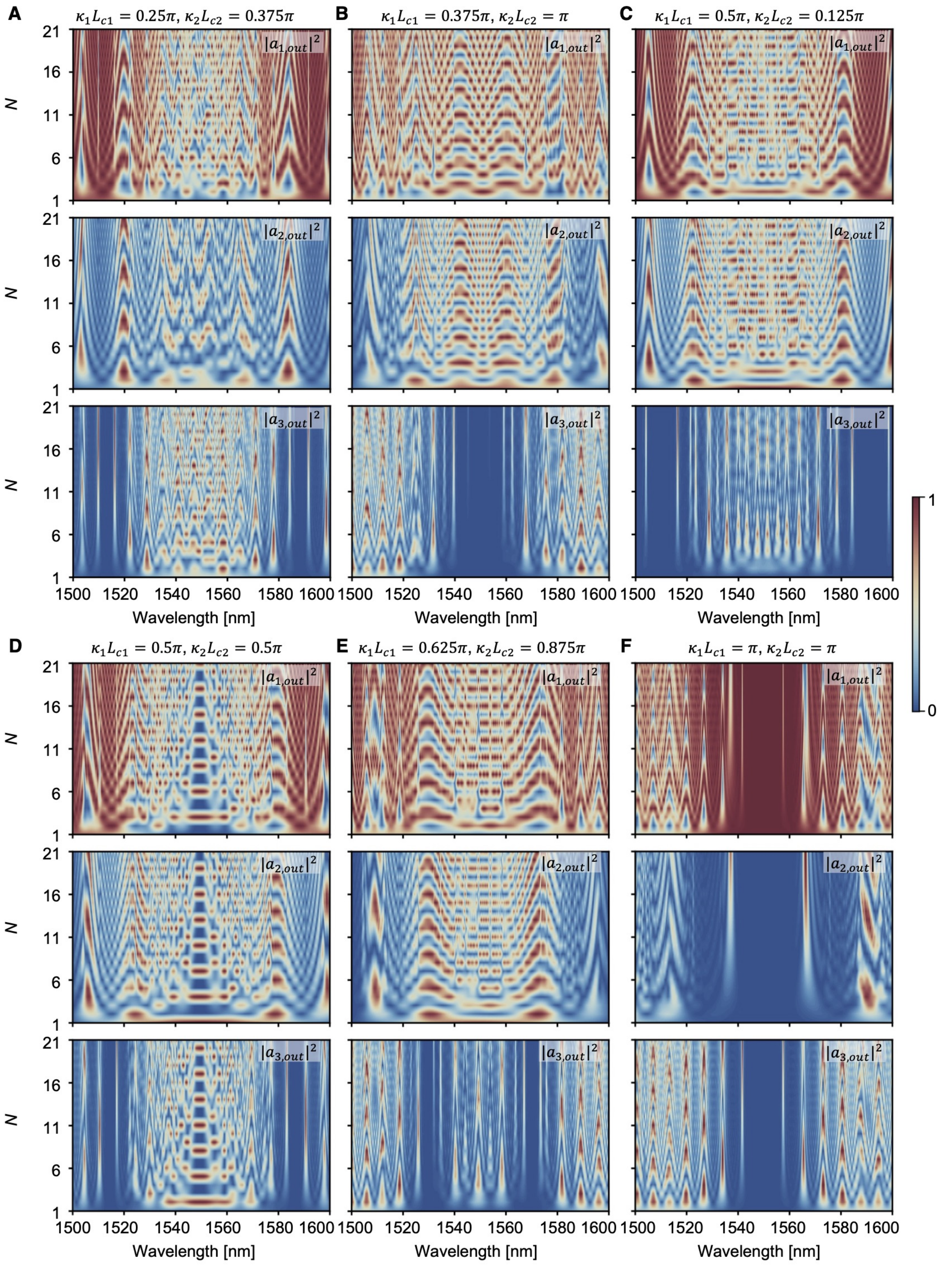

Figure S21: (Caption next page.)

Figure S21: **(Previous page.) Theoretical calculations of the cascaded-mode interferometer with three modes when varying the number of TMCs.** The TE_0, TE_1, and TE_2 modes are used, whose output spectra are noted as $\left|a_{1,\text{out}}\right|^2$, $\left|a_{2,\text{out}}\right|^2$, and $\left|a_{3,\text{out}}\right|^2$, respectively. The effective indexes $n_{\text{eff1}}, n_{\text{eff2}}$, and n_{effi3} of these three modes are 2.74, 2.47, and 1.98, respectively. The grating lengths L_{c1} and L_{c2} are 96 μm and 64 μm, respectively. The gap between the neighboring transmissive mode converters (L_{gap}) is 250 μm. The grating periods of TMCs for the mode conversion between the TE_0 and TE_1 and between TE_0 and TE_2 are 6023 nm and 2126 nm, respectively. We input the TE_0 mode. (**A**) $\kappa_1 L_{c1} = 0.25\pi, \kappa_2 L_{c2} = 0.375\pi$. (**B**) $\kappa_1 L_{c1} = 0.375\pi, \kappa_2 L_{c2} = \pi$. (**C**) $\kappa_1 L_{c1} = 0.5\pi, \kappa_2 L_{c2} = 0.125\pi$. (**D**) $\kappa_1 L_{c1} = 0.5\pi, \kappa_2 L_{c2} = 0.5\pi$. (**E**) $\kappa_1 L_{c1} = 0.625\pi, \kappa_2 L_{c2} = 0.875\pi$. (**F**) $\kappa_1 L_{c1} = \pi, \kappa_2 L_{c2} = \pi$.

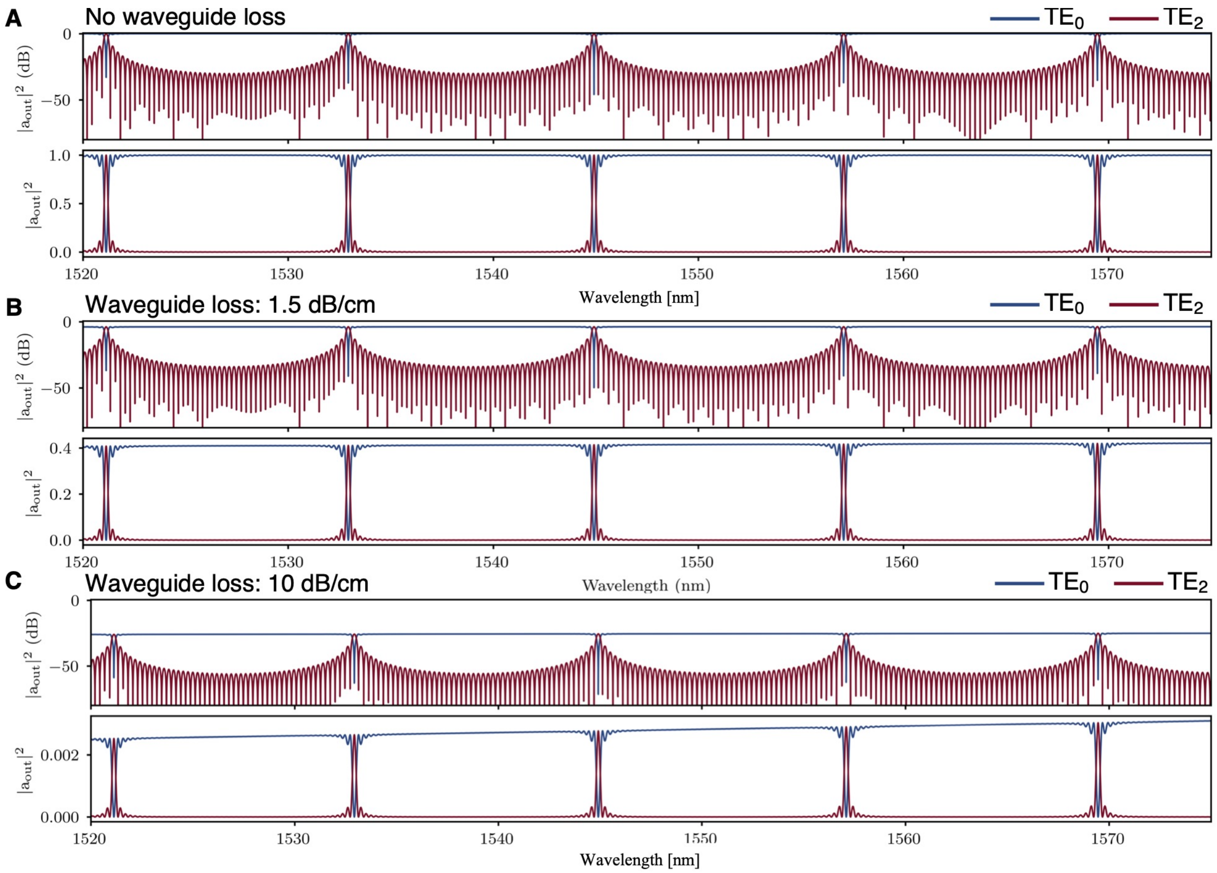

Figure S22: **Loss-independent narrow linewidth spectra of the cascaded-mode interferometers.** The cascaded-mode interferometer consists of 50 mode converters and two waveguide modes (TE_0 and TE_2). The gap distance between the neighboring mode converters is $L_{\text{gap}} = 250\ \mu$m. The coupling strength of each mode converter is $\kappa L_c = \frac{\pi}{2N} = \frac{\pi}{100}$. We input TE_0 mode into this cascaded-mode interferometer. (**A-C**) Calculated output power spectra of the TE_0 and TE_2 modes when we consider the multimode waveguide is lossless (A), with a loss of 1.5 dB/cm (B), and with a loss of 10 dB/cm (C). The free spectral range and the linewidth of the output spectra of this cascaded-mode interferometer are unchanged when the loss is introduced into the waveguide. Only the overall amplitude of the output spectra is reduced when the loss increases. The finesse of the output spectra, which is defined as the ratio of the FSR and the linewidth, is all equal to around 50 for these three cases.

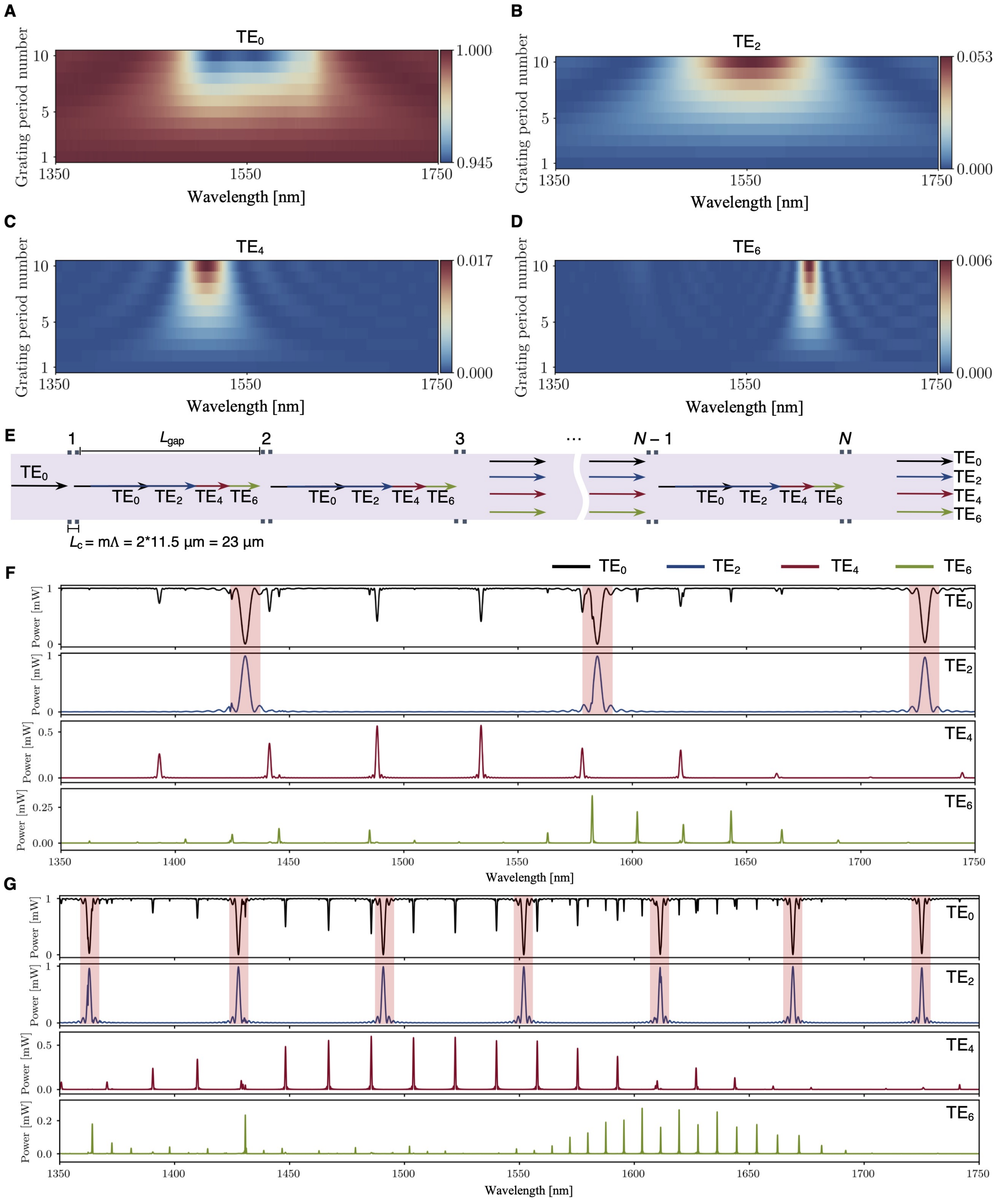

Figure S23: **(Caption next page.)**

Figure S23: **(Previous page.) Simulations of a cascaded-mode interferometer with 34 TMCs.** (**A-D**) Simulated output power spectra of the modes TE_0 (A), TE_2 (B), TE_4 (B), and TE_6 (D) vary with grating period number when the TE_0 mode is input to a single TMC. The TMC is a multimode waveguide with a width of 2500 nm and corrugated with gratings with a period (Λ) of 11.5 μm and a corrugation depth of 20 nm. The grating period is chosen to satisfy the phase matching of mode conversion between TE_0 and TE_2 modes at the central wavelength of 1550 nm. However, a smaller portion of mode conversions also happens between TE_0 and TE_4 modes, and between TE_0 and TE_6 modes at central wavelengths of 1505 nm and 1615 nm, respectively. The coupling coefficients between TE_0 and TE_2 (κ_1), between TE_0 and TE_4 (κ_2), and between TE_0 and TE_6 (κ_3) are estimated as 2.02×10^{-3} μm^{-1}, 1.13×10^{-3} μm^{-1}, and 6.74×10^{-4} μm^{-1}, respectively. (**E**) Schematic of a cascaded-mode interferometer with 34 TMCs, with every TMC having gratings for only two periods. In this case, it satisfies $\kappa_1 L_c = 2\kappa_1 \Lambda = \frac{0.5\pi}{N}$, where $N = 34$. (**F, G**) Simulated power spectra of the modes TE_0, TE_2, TE_4, and TE_6 at the output of the cascaded-mode interferometer when L_{gap} = 100 μm (F) and L_{gap} = 250 μm (G). The TE_0 mode is input to the cascaded-mode interferometer with 1 mW power at each wavelength. The free spectra ranges of the power spectra of the mode TE_2, TE_4, and TE_6 are 148.7 nm, 43.9 nm, and 20.5 nm in (F), and 60.4 nm, 18.3 nm, and 8.4 nm in (G), respectively. The linewidth of the power spectra of the modes TE_2, TE_4, and TE_6 are 3.6 nm, 1.2 nm, and 0.57 nm in (F), and 1.45 nm, 0.46 nm, and 0.21 nm in (G), respectively. Note that the TE_2 mode has a power at spectrum peaks near 1 mW. However, the power at spectrum peaks of TE_4 and TE_6 modes is smaller than 1 mW. It is because $\kappa_1 L_c = \frac{0.5\pi}{N} = 0.046$ while $\kappa_2 L_c = 0.026 < \frac{0.5\pi}{N} = 0.046$ and $\kappa_3 L_c = 0.016 < \frac{0.5\pi}{N} = 0.046$ ($N = 34$). The power at the spectrum peaks can be calculated as $P_{peak} = \sin^2(\kappa m N \Lambda)$, where m is the grating period number in a TMC and N is the number of TMCs.

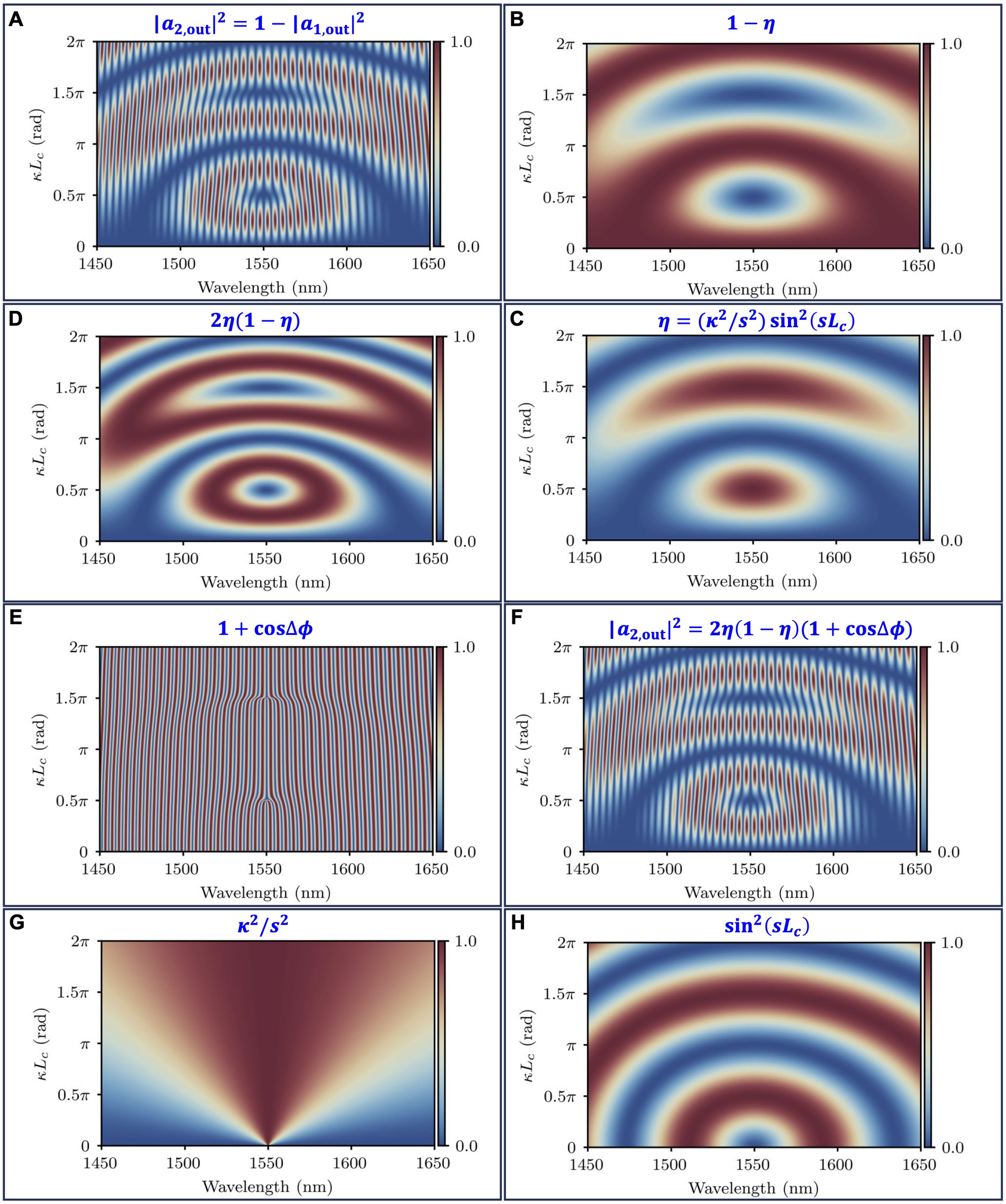

Figure S24: **(Caption next page.)**

Figure S24: **(Previous page.) Origins of doughnut-shaped and fork-shaped structures in Fig. 2B.** (**A**) Calculated output power spectra of the TE_2 modes for varying coupling strength using Eq. 5 and $|a_{2,\text{out}}|^2 = 1 - |a_{1,\text{out}}|^2$. (**B-E**) The power spectra map calculated using $1 - \eta$ (B), η (C), $2\eta(1-\eta)$ (D), and $1+\cos\Delta\phi$ (E), which are factors of Eq. S11. (**F**) Calculated output power spectra of the TE_2 modes using Eq. S11. (**G, H**) The power spectra calculated using $\frac{\kappa^2}{s^2}$ (G) and $\sin^2(sL_c)$ (H), which are factors of η. The doughnut-shaped and fork-shaped structures originate from the components $2\eta(1-\eta)$ and $1+\cos\Delta\phi$ of Eq. S11, respectively.

REFERENCES AND NOTES

1. J. Heritage, A. Weiner, R. Thurston, Picosecond pulse shaping by spectral phase and amplitude manipulation. *Opt. Lett.* **10**, 609–611 (1985).

2. A. M. Weiner, J. P. Heritage, E. Kirschner, High-resolution femtosecond pulse shaping. *J. Opt. Soc. Am. B* **5**, 1563–1572 (1988).

3. A. M. Weiner, Femtosecond optical pulse shaping and processing. *Prog. Quantum Electron.* **19**, 161–237 (1995).

4. J. Chou, Y. Han, B. Jalali, Adaptive RF-photonic arbitrary waveform generator. *IEEE Photonics Technol. Lett.* **15**, 581–583 (2003).

5. S. T. Cundiff, A. M. Weiner, Optical arbitrary waveform generation. *Nat. Photonics* **4**, 760–766 (2010).

6. J. Ye, L. Yan, W. Pan, B. Luo, X. Zou, A. Yi, S. Yao, Photonic generation of triangular-shaped pulses based on frequency-to-time conversion. *Opt. Lett.* **36**, 1458–1460 (2011).

7. Y. Cao, E. Sahin, J. W. Choi, P. Xing, G. F. Chen, D. Ng, B. J. Eggleton, D. T. Tan, Thermo-optically tunable spectral broadening in a nonlinear ultra-silicon-rich nitride Bragg grating. *Photonics Res.* **9**, 596–604 (2021).

8. C. Schnébelin, J. Azaña, H. Guillet de Chatellus, Programmable broadband optical field spectral shaping with megahertz resolution using a simple frequency shifting loop. *Nat. Commun.* **10**, 4654 (2019).

9. M. H. Khan, H. Shen, Y. Xuan, L. Zhao, S. Xiao, D. E. Leaird, A. M. Weiner, M. Qi, Ultrabroad-bandwidth arbitrary radiofrequency waveform generation with a silicon photonic chip-based spectral shaper. *Nat. Photonics* **4**, 117–122 (2010).

10. J. Yao, Arbitrary waveform generation. *Nat. Photonics* **4**, 79–80 (2010).

11. W. Zhang, J. Yao, Photonic integrated field-programmable disk array signal processor. *Nat. Commun.* **11**, 406 (2020).

12. P. Ghelfi, F. Laghezza, F. Scotti, G. Serafino, A. Capria, S. Pinna, D. Onori, C. Porzi, M. Scaffardi, A. Malacarne, V. Vercesi, E. Lazzeri, F. Berizzi, A. Bogoni, A fully photonics-based coherent radar system. *Nature* **507**, 341–345 (2014).

13. D. Marpaung, C. Roeloffzen, R. Heideman, A. Leinse, S. Sales, J. Capmany, Integrated microwave photonics. *Laser Photonics Rev.* **7**, 506–538 (2013).

14. J. S. Fandiño, P. Muñoz, D. Doménech, J. Capmany, A monolithic integrated photonic microwave filter. *Nat. Photonics* **11**, 124–129 (2017).

15. D. Marpaung, J. Yao, J. Capmany, Integrated microwave photonics. *Nat. Photonics* **13**, 80–90 (2019).

16. Y. Liu, A. Choudhary, D. Marpaung, B. J. Eggleton, Integrated microwave photonic filters. *Adv. Opt. Photonics* **12**, 485–555 (2020).

17. X. Fan, I. M. White, S. I. Shopova, H. Zhu, J. D. Suter, Y. Sun, Sensitive optical biosensors for unlabeled targets: A review. *Anal. Chim. Acta* **620**, 8–26 (2008).

18. J. Albert, L.-Y. Shao, C. Caucheteur, Tilted fiber Bragg grating sensors. *Laser Photonics Rev.* **7**, 83–108 (2013).

19. Y. Tan, L.-P. Sun, L. Jin, J. Li, B.-O. Guan, Microfiber Mach-Zehnder interferometer based on long period grating for sensing applications. *Opt. Express* **21**, 154–164 (2013).

20. N. N. Klimov, S. Mittal, M. Berger, Z. Ahmed, On-chip silicon waveguide Bragg grating photonic temperature sensor. *Opt. Lett.* **40**, 3934–3936 (2015).

21. D. Monzon-Hernandez, A. Martinez-Rios, I. Torres-Gomez, G. Salceda-Delgado, Compact optical fiber curvature sensor based on concatenating two tapers. *Opt. Lett.* **36**, 4380–4382 (2011).

22. A. Li, Y. Fainman, On-chip spectrometers using stratified waveguide filters. *Nat. Commun.* **12**, 2704 (2021).

23. K. U. Schreiber, J. Kodet, U. Hugentobler, T. Klügel, J.-P. R. Wells, Variations in the Earth's rotation rate measured with a ring laser interferometer. *Nat. Photonics* **17**, 1054–1058 (2023).

24. I. Newton, A new theory about light and colors. *Am. J. Phys.* **61**, 108–112 (1993).

25. H. Y. Choi, K. S. Park, S. J. Park, U.-C. Paek, B. H. Lee, E. S. Choi, Miniature fiber-optic high temperature sensor based on a hybrid structured Fabry–Perot interferometer. *Opt. Lett.* **33**, 2455–2457 (2008).

26. W. Zhang, J. Yao, A fully reconfigurable waveguide Bragg grating for programmable photonic signal processing. *Nat. Commun.* **9**, 1396 (2018).

27. S.-J. Kim, T.-J. Eom, B. H. Lee, C.-S. Park, Optical temporal encoding/decoding of short pulses using cascaded long-period fiber gratings. *Opt. Express* **11**, 3034–3040 (2003).

28. H. A. Macleod, H. A. Macleod, *Thin-Film Optical Filters* (CRC Press, 2010).

29. C. Yang, J. Wen, X. Chen, H. Luo, Y. Zhu, H. Wang, T. Zheng, Y. Zhang, W. Shen, Wavelength-selective light trapping with nanometer-thick metallic coating. *Adv. Photonics Res.* **3**, 2100338 (2022).

30. C. Wan, D. Woolf, C. M. Hessel, J. Salman, Y. Xiao, C. Yao, A. Wright, J. M. Hensley, M. A. Kats, Switchable induced-transmission filters enabled by vanadium dioxide. *Nano Lett.* **22**, 6–13 (2021).

31. P. Cheben, J. H. Schmid, A. Delâge, A. Densmore, S. Janz, B. Lamontagne, J. Lapointe, E. Post, P. Waldron, D.-X. Xu, A high-resolution silicon-on-insulator arrayed waveguide grating microspectrometer with sub-micrometer aperture waveguides. *Opt. Express* **15**, 2299–2306 (2007).

32. X. Gu, Wavelength-division multiplexing isolation fiber filter and light source using cascaded long-period fiber gratings. *Opt. Lett.* **23**, 509–510 (1998).

33. Y. Geng, X. Li, X. Tan, Y. Deng, Y. Yu, In-line flat-top comb filter based on a cascaded all-solid photonic bandgap fiber intermodal interferometer. *Opt. Express* **21**, 17352–17358 (2013).

34. W. S. Mohammed, P. W. Smith, X. Gu, All-fiber multimode interference bandpass filter. *Opt. Lett.* **31**, 2547–2549 (2006).

35. J. E. Antonio-Lopez, A. Castillo-Guzman, D. A. May-Arrioja, R. Selvas-Aguilar, P. LiKamWa, Tunable multimode-interference bandpass fiber filter. *Opt. Lett.* **35**, 324–326 (2010).

36. J. Wu, T. Moein, X. Xu, D. J. Moss, Advanced photonic filters based on cascaded Sagnac loop reflector resonators in silicon-on-insulator nanowires. *APL Photonics* **3**, 046102 (2018).

37. R. A. Cohen, O. Amrani, S. Ruschin, Response shaping with a silicon ring resonator via double injection. *Nat. Photonics* **12**, 706–712 (2018).

38. V. Ivakhnenko, S. N. Shevchenko, F. Nori, Nonadiabatic Landau–Zener–Stückelberg–Majorana transitions, dynamics, and interference. *Phys. Rep.* **995**, 1–89 (2023).

39. T. Suzuki, H. Nakazato, Generalized adiabatic impulse approximation. *Phys. Rev. A* **105**, 022211 (2022).

40. V. Ginis, M. Piccardo, M. Tamagnone, J. Lu, M. Qiu, S. Kheifets, F. Capasso, Remote structuring of near-field landscapes. *Science* **369**, 436–440 (2020).

41. A. Yariv, P. Yeh, A. Yariv, *Photonics: Optical Electronics in Modern Communications*, vol. 6 (Oxford Univ. Press, 2007).

42. W.-P. Huang, Coupled-mode theory for optical waveguides: An overview. *J. Opt. Soc. Am. A* **11**, 963–983 (1994).

43. V. Ginis, I.-C. Benea-Chelmus, J. Lu, M. Piccardo, F. Capasso, Resonators with tailored optical path by cascaded-mode conversions. *Nat. Commun.* **14**, 495 (2023).

44. B. H. Lee, Y. H. Kim, K. S. Park, J. B. Eom, M. J. Kim, B. S. Rho, H. Y. Choi, Interferometric fiber optic sensors. *Sensors* **12**, 2467–2486 (2012).

45. L.-M. Needham, C. Saavedra, J. K. Rasch, D. Sole-Barber, B. S. Schweitzer, A. J. Fairhall, C. H. Vollbrecht, S. Wan, Y. Podorova, A. J. Bergsten, B. Mehlenbacher, Z. Zhang, L. Tenbrake, J. Saimi, L. C. Kneely, J. S. Kirkwood, H. Pfeifer, E. R. Chapman, R. H. Goldsmith, Label-free detection and profiling of individual solution-phase molecules. *Nature* **629**, 1062–1068 (2024).

46. S. Yu, J. Lu, V. Ginis, S. Kheifets, S. W. D. Lim, M. Qiu, T. Gu, J. Hu, F. Capasso, On-chip optical tweezers based on freeform optics. *Optica* **8**, 409–414 (2021).

47. J. Lu, V. Ginis, S. W. D. Lim, F. Capasso, Helicity and polarization gradient optical trapping in evanescent fields. *Phys. Rev. Lett.* **131**, 143803 (2023).

48. C. Jiang, K. Zhou, B. Sun, Y. Wan, Y. Ma, Z. Wang, Z. Zhang, C. Mou, Y. Liu, Multiple core modes conversion using helical long-period fiber gratings. *Opt. Lett.* **48**, 2965–2968 (2023).

49. A. Mohanty, M. Zhang, A. Dutt, S. Ramelow, P. Nussenzveig, M. Lipson, Quantum interference between transverse spatial waveguide modes. *Nat. Commun.* **8**, 14010 (2017).

50. G. Weihs, M. Reck, H. Weinfurter, A. Zeilinger, All-fiber three-path Mach–Zehnder interferometer. *Opt. Lett.* **21**, 302–304 (1996).

51. A. Crespi, R. Osellame, R. Ramponi, D. J. Brod, E. F. Galvao, N. Spagnolo, C. Vitelli, E. Maiorino, P. Mataloni, F. Sciarrino, Integrated multimode interferometers with arbitrary designs for photonic boson sampling. *Nat. Photonics* **7**, 545–549 (2013).

52. B. Seron, L. Novo, N. J. Cerf, Boson bunching is not maximized by indistinguishable particles. *Nat. Photonics* **17**, 702–709 (2023).

53. H. Zhou, J. Dong, J. Cheng, W. Dong, C. Huang, Y. Shen, Q. Zhang, M. Gu, C. Qian, H. Chen, Z. Ruan, X. Zhang, Photonic matrix multiplication lights up photonic accelerator and beyond. *Light Sci. Appl.* **11**, 30 (2022).

54. H. H. Zhu, J. Zou, H. Zhang, Y. Z. Shi, S. B. Luo, N. Wang, H. Cai, L. X. Wan, B. Wang, X. D. Jiang, J. Thompson, X. S. Luo, X. H. Zhou, L. M. Xiao, W. Huang, L. Patrick, M. Gu, L. C. Kwek, A. Q. Liu, Space-efficient optical computing with an integrated chip diffractive neural network. *Nat. Commun.* **13**, 1044 (2022).

55. X. Meng, G. Zhang, N. Shi, G. Li, J. Azaña, J. Capmany, J. Yao, Y. Shen, W. Li, N. Zhu, M. Li, Compact optical convolution processing unit based on multimode interference. *Nat. Commun.* **14**, 3000 (2023).

56. Z. Du, K. Liao, T. Dai, Y. Wang, J. Gao, H. Huang, H. Qi, Y. Li, X. Wang, X. Su, X. Wang, Y. Yang, C. Lu, X. Hu, Q. Gong, Ultracompact and multifunctional integrated photonic platform. *Sci. Adv.* **10**, eadm7569 (2024).

57. T. H. Fay, P. Hendrik Kloppers, The Gibbs' phenomenon. *Int. J. Math. Educ. Sci. Technol.* **32**, 73–89 (2001).
